# Urinary specific gravity as an alternative for the normalisation of endocrine metabolite concentrations in giant panda (*Ailuropoda melanoleuca*) reproductive monitoring

**DOI:** 10.1371/journal.pone.0201420

**Published:** 2018-07-26

**Authors:** Jella Wauters, Kirsten S. Wilson, Tim Bouts, Iain Valentine, Koen Vanderschueren, Cyrillus Ververs, A. Forbes Howie, Mick T. Rae, Ann Van Soom, Rengui Li, Desheng Li, Hemin Zhang, Lynn Vanhaecke

**Affiliations:** 1 Laboratory of Chemical Analysis, Department of Veterinary Public Health and Food Safety, Faculty of Veterinary Medicine, Ghent University, Merelbeke, Belgium; 2 Pairi Daiza – Pairi Daiza Foundation, Brugelette, Belgium; 3 MRC Centre for Reproductive Health, University of Edinburgh, Edinburgh, United Kingdom; 4 Royal Zoological Society of Scotland, Edinburgh Zoo, Edinburgh, United Kingdom; 5 Department of Reproduction, Obstetrics and Herd Health, Faculty of Veterinary Medicine, Ghent University, Merelbeke, Belgium; 6 School of Applied Sciences, Edinburgh Napier University, Sighthill Campus, Edinburgh, United Kingdom; 7 China Conservation and Research Centre for Giant Panda (CCRCGP), DuJiangYan City, SiChuan Province, China; Sichuan University, CHINA

## Abstract

Reproductive monitoring for captive breeding in giant pandas is based on behavioural observation and non-invasive hormone analysis. In urine, interpretation of results requires normalisation due to an animal’s changing hydration. Correction of urinary concentrations based on creatinine is the gold standard. In this study, a largely unexplored, easy-to-perform normalisation technique, based on urinary specific gravity (USpG), was examined and compared to creatinine. To this extent, six cycles from two female pandas (SB741(1) and SB569(5)) were monitored through urine analysis for oestrogen, progesterone, ceruloplasmin and 13,14-dihydro-15-keto-PGF2a (PGFM). The Pearson’s correlation between creatinine and USpG was high (r = 0.805–0.894; p < 0.01), indicative for a similar performance of both normalisation methods. However, generally lower values were observed during pro-oestrus and primary (progesterone) rise. This could be associated with huge shifts in appetite, monitored by faecal output (kg) with an averaged > 50% decrease during oestrus and >50% increase during primary progesterone rise. In parallel, respectively highest and lowest creatinine and USpG levels, were measured, with creatinine obviously more affected as a result of linkage with muscle tissue metabolism affected by reproductive hormones. As a consequence, metabolite levels were significantly different between both corrected datasets with significantly higher oestrogen peak levels during oestrus ranging from 2.13–86.93 and 31.61–306.45 ng/mL (USpG correction) versus 2.33–31.20 and 36.36–249.05 ng/mL Cr (creatinine correction) for SB569 and SB741 respectively, and significant lower progesterone levels during primary progesterone rise ranging from 0.35–3.21 and 0.85–6.80 ng/mL (USpG correction) versus 0.52–10.31 and 2.10–272.74 ng/mL Cr (creatinine correction) for SB569 and SB741 respectively. Consequently, USpG correction rendered unbiased profiles, less subject to variation and metabolic artefacts and therefore allowed a more straightforward identification of peak oestrogen and onset of secondary progesterone rise, being potentially advantageous for future studies unravelling key giant panda reproductive events, including (delayed) implantation. The alternative application of USpG as a normalisation factor was further supported by its easy application and environmental and technical robustness.

## Introduction

Currently, the biggest threats to a self-sustaining wild population of the giant panda (*Ailuropoda melanoleuca*), previously endangered and more recently listed as vulnerable on the IUCN red-list, are restriction, degradation and fragmentation of its habitat [1, 2]. Additionally, population growth is confined by specific reproductive features: the annual mating season is limited to a short single oestrus, litters have a restricted size and a prolonged interbirth period is generally maintained in the wild [3, 4]. The reproductive cycle of the giant panda is, similar to most ursids, characterized by seasonality, delayed implantation and the occurrence of pseudopregnancy. Unlike other bears, the giant panda is a mono-oestrous species with a brief oestrus, restricting the period of time for natural mating or artificial insemination (AI) to 1 to 3 days per year [3, 4]. The physiological changes associated with the different stages of the reproductive process are reflected in the behaviour and endocrine status of the animal. Gathering this information, through observation and monitoring, has been key in understanding and predicting the reproductive biological processes [5, 6].

(Pro)oestrus is announced by behavioural changes such as frequent urination, scent marking and depressed appetite, and includes lordosis and increasing vocalizations closer to peak oestrogen values [7]. A crescendo of oestrus behaviour is reflected in rising oestrogen levels, which may start to increase from baseline levels about two weeks prior to the peak oestrogen value preceding ovulation. The fertility window is currently defined as the 48 hours following the peak oestrogen value, with natural mating and/or AI performed once or twice during this period [7]. The luteal phase follows shortly after the oestrogen peak. A modest primary increase in progesterone can be identified during the *corpus luteum* dormancy phase, persisting for several months, which is followed by a shorter, strongly pronounced secondary progesterone rise. At the end of this active luteal phase, progesterone returns to baseline, corresponding with the birth of the cub, if not lost earlier, or with the end of pseudopregnancy. Despite their widespread use for monitoring a potential pregnancy, the progesterone levels, as well as the related behaviour, fail to discriminate between pregnancy, pseudopregnancy and lost pregnancy [8]. Therefore, several research groups have embarked in searching for other pregnancy biomarkers, known to be effective in other (predominantly domestic) animal species. A urinary profile of the acute phase protein ceruloplasmin (CP), which is increased in serum during human and canine pregnancies, was recently described in the giant panda for the detection of successful conception, as well as for the monitoring of successful and lost pregnancies [9–11]. A recent study strongly suggests the potential of 13,14-dihydro-15-keto-PGF_2α_ (PGFM) as a biomarker to discriminate pregnancy from pseudopregnancy, with predictive capacities towards a birth date, both on the long (3 weeks) and short term (24-48h prior to birth) [12, 13]. PGFM is the excreted metabolite of PGF_2α_, a natural luteolysin secreted by the endometrium in several species [14, 15]. In the dog, however, synthesis of PGF_2α_ occurs in the placenta and is upregulated after implantation with pre-partum peak levels [16, 17]. In ongoing studies, the biomarker indeed confirmed to be indicative for early pregnancy and allowed prediction of the timing of presumable birth accurately [18]. Relaxin, produced by the placenta, corpus luteum, endometrium and some non-reproductive organs has been evaluated as a biomarker for pregnancy in endangered species [19, 20]. However, due to confounding factors such as delayed implantation, pseudopregnancy and presumably frequent foetal absorptions, results have been inconclusive in the giant panda, in contrast to the domestic dog.

In this study, we monitored the reproductive cycle of two female giant panda through the analysis of urinary oestrogens, progesterone, ceruloplasmin and PGFM by enzymatic immuno-assays. Since urine metabolite concentrations are influenced by the hydration status of the animal, a corrective factor should be included in data analysis. This is usually performed based on creatinine correction. Creatinine is freely filtered by the kidney from the blood stream into urine and routinely used as a measure of urine concentration. The popularity of the use of creatinine as a correction factor in urine can be attributed to the fact that the daily excretion of creatinine in individuals has been accepted as fairly consistent [21–23]. However, creatinine is a turnover product of the muscle tissue metabolism and, more recently, its concentration in serum or urine has been demonstrated as strongly influenced by several factors including acute infection, injury, stress, diet and extreme exercise [22–25]. Several of the latter factors (e.g. stress, diet, activity rate) are well known to be influenced by sex hormones being released in high concentrations during specific stages of the reproductive cycle e.g. during (pro)oestrus and (pseudo)pregnancy [26–28]. In contrast to previously reported studies on giant panda reproductive biology, we therefore investigated the use of urinary specific gravity (USpG) as a corrective measure for variable concentrations of urine samples. USpG is the ratio of the density of a urine specimen to the density of water. Diet type and fasting status are also described as interacting factors for USpG, but their impact is acknowledged to be minimal in comparison to that of creatinine [29]. Despite being a direct measure of urinary concentration, its application as a correction factor for urine metabolites has thus far not been explored in depth.

## Material and methods

### Sample collection

Samples were collected from two female pandas. Hao Hao (SB741; ° 7 July 2009), resident at Pairi Daiza, Brugelette, Belgium since 2014 and Tian Tian (SB569; °24 August 2003), resident at Edinburgh Zoo, Edinburgh, Scotland, UK since 2011, were included in the study with 1 successfully bred (2016) and 5 non-successfully bred (2013–2017) cycles respectively (artificial insemination was performed for all cycles following peak oestrogen).

Urine was aspirated from the ground with a clean syringe and transferred to a collection vial. Sample collection was pursued daily at least from pro-oestrus until parturition or end of cycle. During the pro-oestrus and the presumed prepartum period, numerous samples were gathered throughout the day and immediately analysed at the zoos’ facilities (Pairi Daiza and Edinburgh Zoo). All other samples were stored at -20°C after sampling until analysis at the laboratories (Laboratory of Chemical Analysis and the Queens’s Medical Research Institute).

In parallel, daily monitoring of faecal output (both pandas) and bodyweight (Tian Tian only) was part of the study, however, bodyweight records were missing particularly during secondary progesterone rise due to behavioural changes.

This research was solely performed based on non-invasive sampling of urine as a waste product (aspiration from the ground in absence of the animal during routine maintenance of the enclosure) and therefore was not affecting animal welfare. Body weighing was performed during daily routine monitoring of the animal’s health (zoo management) and these data were retrospectively acquired for our study. As a result, all animal-related work has been conducted according to relevant national and international guidelines and no specific ethical approval was mandatory for this study.

### Procedures and method validity

#### Sample pre-treatment

Fresh or defrosted urine was transferred to a 15 mL Nunc^®^ tube (Thermo Scientific) through a small-sized disposable plastic funnel with cotton-wool filter plug allowing filtering to clean-up the urine sample prior to analysis. Samples were aliquoted and banked at -20°C for future research purposes.

#### Urinary specific gravity

The urinary specific gravity (USpG) was determined by a handheld digital refractometer (Pal-USG (Cat), Atago^™^, Fukaya, Japan). The instrument was calibrated with 300 μL ultra-pure water before measuring the first sample (300 μL), according to manufacturers’ guidelines. The instrument was cleaned with ultra-pure water between samples. The robustness of the technique was additionally evaluated by investigating potential technical and environmental conditions. Therefore, two differently scaled refractometers were compared measuring 350 giant panda urine samples (Pal-10S (Human) and Pal-USG (Cat) (Atago^™^, Fukaya, Japan)). The impact of 3 freeze-thaw cycles was investigated as well as the influence of 3 different sample and environment temperatures (4, 20 and 37°C) (n = 20). Additionally, the minimum sample volume for reliable read-out was determined by challenging 10 different sample volumes (300–250–200–150–100–90–80–70–60–50 μL) on 30 samples.

Urinary biomarker levels were corrected based on the formula described by Miller et al. (2004) [22], with USpG corrected concentration = raw hormone concentration x [(USpG _target_ -1.000)/ (USpG _sample_− 1.000)]. USpG target represents the population mean USpG for giant panda urine. As no details were available for this species, the mean USpG was estimated based on minimum 30 different urine specimens from each female panda (with normal daily behaviour and no signs of upcoming oestrus). This resulted in a mean USpG of 1.005 (± 0.002)_SB741_ and 1.008 (± 0.002)_SB569_.

#### Creatinine

For samples of Tian Tian, quantitative creatinine analysis was undertaken using a commercial Creatinine Enz Liquid Kit (Sentinel Diagnostics, Milan, Italy) on a Cobas Fara centrifugal analyser (Roche Diagnostics Ltd., UK) by means of an enzymatic colorimetric determination of creatinine following the manufacturer’s guidelines. Urine samples were diluted 10 or 20 times on a MicroLab Diluter (MicroLab 500, Hamilton Company, USA) prior to analysis.

For Hao Hao’s samples, quantitative creatinine analysis was performed based on the multispecies Ncal^™^ International Standard DetectX^®^ urinary creatinine detection kit (K002-H1, Arbor Assays Ann Arbor, Michigan, USA). Prior to sample analysis, dilution factors were optimized through a selection of seven samples covering the range of USpG to be expected (between 1.002 and 1.018). The latter samples were analysed comparing different dilution factors (i.e. 2, 5, 10, 20, 40). Sample analysis was performed following the manufacturer’s guidelines. The optical density was determined by the Emax Plus Microplate Reader (Molecular Devices, LCC, Sunnyvale, California, US) at 490 nm. Creatinine concentrations were calculated based on a four-parameter logistic curve fit (4PLC) (SoftMaxPro Software, version 6.4.1, Molecular Devices, LCC, Sunnyvale, California, US) after subtracting the mean ODs for the blank and multiplying by the dilution factor.

An inter-laboratory coefficient of variation was determined on a batch of 85 samples analysed with both techniques (CV = 7.76%).

Correction based on urinary creatinine levels was performed by taking the ratio between raw biomarker concentrations and the creatinine level expressed in mg/mL.

#### Oestrogens

Oestrogen-monitoring was performed multiple times a day following pre-oestrous behavioural changes until ovulation. After insemination, samples were analysed retrospectively in larger sample batches.

For Hao Hao’s samples, analysis of oestrogens was performed with the DetectX^®^ Estrone Enzyme Immunoassay Kit (K031-H1, Arbor Assays^™^, Ann Arbor, Michigan, USA). However, the oestrone standard was replaced by the oestrone-3-sulphate (E1S) standard (C135-125 μL, Arbor Assays^™^, Ann Arbor, Michigan, USA). Cross reactivity of 100%, 112%, 65.5% and 5% is reported for oestrone, oestrone-3-glucuronide, oestrone-3-sulphate (E1S) and oestradiol respectively. For Tian Tian’s samples, analysis was performed with the DetectX^®^ oestrone-3-glucuronide (E1G) Enzyme Immunoassay Kit (K036-H5, Arbor Assays^™^, Ann Arbor, Michigan, USA). Cross reactivity of 100%, 66.6%, 238%, 7.8%, 3.8% and 3.3% is reported for oestrone-3-glucuronide, oestrone-3-sulphate, oestrone, 17β-oestradiol, oestradiol-3-glucuronide and oestradiol-3-sulphate respectively. The kits were used following the recommendations of the manufacturer. Prior to sample dilution, kit-specific reagents were prepared. Assay buffer was five times diluted in ultra-pure water (and used for dilution of samples and standards), while wash buffer required a 1:20 dilution. The dilution of the samples was dependent on the reproductive status of the animal; 1:10 dilution was sufficient for baseline levels, whilst a dilution of 1:100 and 1:500 was required during pre-oestrus and at peak oestrogen levels respectively. Sample analysis was performed following the manufacturer’s guidelines. The optical density was determined at 450 nm (Emax Plus Microplate Reader (Molecular Devices, LCC, Sunnyvale, California, US). A standard curve was created based on a 4PLC fitting, after calculating the % B/B_0_ = 100*[(OD _standard_−OD _NSB_)/(OD B0 –OD N_SB_)] (SoftMaxPro Software, version 6.4.1, Molecular Devices, LCC, Sunnyvale, California, US). The obtained concentrations, based on the %B/B0 curve, had to be multiplied by the dilution factor to obtain neat (raw) sample values.

Inter- (between plates) and intra- (within plates) coefficients of variation (CV, %) were determined for the E1S and E1G detection. The intra-coefficients of variation were calculated for two batches of samples for the E1S kit. A smaller batch (n = 32) was analysed between November 2014 and February 2015 under optimal environmental laboratory conditions (CV = 6.02%). A larger batch of samples (n = 87) analysed between February and June 2016 was subjected to varying environmental conditions not always in accordance with the recommendations of the kit (CV = 0.93%). For E1G a CV of 3.97% was achieved based on 36 samples. The inter-coefficient of variation was derived from a sub-batch (n = 14) of the 2016-sample pool for E1S (CV = 10.47%), while for E1G 77 samples were involved (CV = 12.50%).

#### Progesterone

Analyses of progesterone levels were performed on a weekly basis, with real-time daily monitoring from one week before potential birth.

The urinary progesterone levels were determined with the DetectX^®^ progesterone Enzyme Immunoassay Kit (K025-H1, Arbor Assays^™^, Ann Arbor, Michigan, USA) for both giant pandas.

Samples were diluted with the dilution factor dependent of the reproductive status of the animal. This was predominantly a 1:10 dilution except for a 1:100 dilution during the secondary progesterone rise.

The assay’s protocol is very similar to the protocol for oestrogen detection, however kit-specific reagents were used and the optical density was monitored by the Emax Plus Microplate Reader (Molecular Devices, LCC, Sunnyvale, California, US) at 450 nm. The standard curve and sample levels were acquired identically as described for E1S, with 4PLC fitting (SoftMaxPro Software, version 6.4.1, Molecular Devices, LCC, Sunnyvale, California, US).

An intra-assay CV was determined (n = 11; CV = 4.67%).

#### Ceruloplasmin

After artificial insemination, analyses of ceruloplasmin levels were performed on a weekly basis.

For Tian Tian’s samples, analysis was performed through measurement of ferri-oxidase activity using N,N-dimethyl-p-phenylenediamine oxidase as a substrate (DPP; Sigma-Aldrich, UK) as optimised by Sunderman and Nomoto [30] and modified for a Cobas Fara centrifugal analyser. Undiluted urine was analysed, and the presence of ceruloplasmin was indicated by a quantifiable pink/purple colour.

For Hao Hao’s samples ceruloplasmin levels were determined by the DetectX^®^ Ceruloplasmin Colorimetric Activity kit (K035-H1, Arbor Assays^™^, Ann Arbor, Michigan, USA) following the manufacturer’s guidelines.

The optical density was generated from each well with the Emax Plus Microplate Reader (Molecular Devices, LCC, Sunnyvale, California, US) at 560 nm.

A standard curve was created after subtracting the mean OD for the zero standard from each standard OD value and fitting it to a 4PLC model. The sample activity obtained (treating samples identically to standards e.g. correcting based on the zero standard) was multiplied by the dilution factor.

#### PGFM

With the assumed onset of secondary progesterone rise, PGFM-analysis was undertaken twice weekly. In the final week before potential birth, PGFM was monitored in real-time in order to monitor the end terms of the potential pregnancy and to predict the date of birth. PGFM levels during oestrus and primary progesterone rise were investigated retrospectively.

For PGFM monitoring the DetectX^®^ 13,14-dihydro-15-keto-PGF2_α_ (PGFM) Enzyme Immunoassay Kit was applied (K022-H1, Arbor Assays^™^, Ann Arbor, Michigan, USA) and the manufacturer’s guidelines adhered to.

Dilution of samples was also dependent on the animal’s reproductive status, with generally a 1:10 dilution until the start of the secondary rise and a 1:100 dilution during the middle/late secondary rise. At the end of the secondary rise (e.g. a few days prepartum/ end of cycle), two dilution factors (1:100 and 1:500) were explored.

### Data analysis

A preliminary screening of the USpG’s suitability for normalisation was performed by assessing the linear correlation (Pearson’s correlation coefficient, significant if p<0.05) between raw E1S values and the respective USpG in 32 urinary samples from Hao Hao during a non-reproductive period (14 November 2014 till 10 February 2015). Additionally, the correlation between the USpG outcomes obtained with two different refractometers was also determined (Pearson’s correlation coefficient, significant if p<0.05).

Analysis of the reproductive cycles was facilitated by defining reproductive periods, with the peak oestrogen value as the point of reference (Day 0; D_0_). More specifically, 6 periods were defined: the anoestrus, the pro-oestrus period, the post-oestrus period, the primary progesterone rise, the secondary progesterone rise and the post-birth or post-secondary progesterone rise period (Table 1). Birth occurred at D_145_ in SB741 2016. The start of the pro-oestrus period was defined based on changes in the oestrogen/progesterone ratio, while the post-oestrus period included 7 days after peak oestrogen. Onset of the secondary progesterone rise was arbitrarily calculated based on the following formula [mean concentration progesterone _primary progesterone rise_ + 2 x standard deviation _primary progesterone rise_] with at least 2 consecutive days of progesterone concentrations exceeding this value [8]. These reference periods were defined for both the USpG and creatinine corrected datasets (Table 1). However, any statistical data assessment as well as the acquisition of descriptives (mean, standard deviation, median and range) were performed based on the USpG corrected dataset and its respective reproductive period definition. The datasets were explored for linear correlations between USpG, Cr, faecal output and bodyweight, applying Pearson’s correlation coefficient analysis (significance level set at p < 0.05).

**Table 1:** An overview of the reproductive periods expressed in days from peak oestrogen for five cycles (2013–2017) of Tian Tian_SB569_ and one cycle (2016) of Hao Hao_SB741_.

Feature	Tian Tian 2013	Tian Tian 2014	Tian Tian 2015	Tian Tian 2016	Tian Tian 2017	Hao Hao 2016
**Anoestrus**	D-63-D-14	D-107-D-14	D-111-D-13	D-208-D-10	D-104-D-9	No records
**Pro-oestrus**	D-13-D0	D-13-D0	D-12-D0	D-9-D0	D-8-D0	D-8-D0
**Peak oestrogen**	D0	D0	D0	D0	D0	D0
**Postoestrus**	D1-D7	D1-D7	D1-D7	D1-D7	D1-D7	D1-D7
**Primary P4 rise USpG**	D8-D98	D8-D97	D8-D111	D8-D109	D8-D128	D8-D61
**Secondary P4 rise USpG**	D99-D165	D98-D166	D112-D163	D110-D157	D129-D179	D62-D114
**Primary P4 rise Cr**	D8-D79	D8-D79	D8-D91	D8-D98	D8-D113	D8-D77
**Secondary P4 rise Cr**	D80-D165	D80-D166	D92-D163	D99-D157	D114-D179	D78-D114
**Postbirth**	no birth	no birth	no birth	no birth	no birth	D115-D126

P4 = progesterone; USpG = urinary specific gravity; Cr = creatinine

Additionally, metabolites’ concentrations were compared for each reproductive period in each normalised data set. Due to the non-homoscedastic character of the datasets deviating from normality, this was performed through non-parametric data analysis. Paired comparisons were performed between the respective datasets applying the Wilcoxon Signed Rank test (significant if p < 0.05). Within the USpG- and creatinine- datasets respectively, hormone levels were compared between the defined reproductive periods based on the Independent-Samples Kruskall Wallis test with post hoc Dunn’s comparison (significant if p < 0.05). The statistical analyses were performed with SPSS version 25.0 (IBM, Brussels, Belgium).

## Results and discussion

### Rudimentary quality assessments

#### USpG

To evaluate whether USpG was a potential alternative correction factor for urinary concentration, 32 non-reproductive samples of Hao Hao (season 2015) were investigated at the early start of the study. A strong correlation was observed between raw urinary oestrogen concentrations and USpG, being indicative of a promising alternative application of USpG as correction factor for urinary metabolite concentrations (r = 0.925).

A broad range refractometer (1.000–1.080) was selected for purchase since no literature details were available on the urinary density of giant pandas. Giant panda urinary density, however, performed at the lower edge of the range, with average values for Hao Hao and Tian Tian at 1.005 and 1.008, respectively, during anoestrus.

To allow the best consulting towards other researchers, additional experiments were outlined, challenging a differently scaled refractometer on the one hand and evaluating environmental conditions on the other hand. Therefore, a 1.000–1.060 scaled refractometer was tested (Pal-10S (Human), Atago^™^, Fukaya, Japan) in comparison to the Pal-USG (Cat) (Atago^™^, Fukaya, Japan). On a sample batch of 350 giant panda urine samples from both non-active and active reproductive periods, the average USpG value was higher (1.008 ± 0.006) when measured with the human compared to the cat (1.006 ± 0.005) refractometer. Correlation between USpG values acquired by both refractometers was high (r = 0.990), therefore demonstrating no superior performance of one of both compared to the other. However, it is important to highlight that in terms of longitudinal monitoring, it is mandatory to use the same scaled refractometer, and the correlated average value, throughout the whole cycle for adequate correction of urinary metabolite concentrations [31].

To better understand why different refractometers show discrepancies, the mechanism behind this instrument will be briefly explained. Specific gravity is the ratio of the density of a substance (e.g. urine) to the density of water and can be monitored based on refractometry [32]. A refractometer basically reports outcomes based on refractive indexes of a ray of light when it strikes a boundary between one medium (e.g. air) to another (e.g. urine). This physical characteristic is described by the Snell-Descartes law which allows calculating the refractive index of an unknown second medium [33]. Refractometry is affected by number, mass and chemical structure of the dissolved particles. In urine, it is largely influenced by albumin, creatinine, glucose, hemoglobin, sodium chloride and urea [32, 34]. Using this information, specific scales are set up by manufacturing companies to render different species-specific USpG refractometers with each their own conversion formula derived from the refractive index and taking into account the ratio with water. Additionally, the measurement of the refractive index also depends on the temperature and wavelength of the light. While the wavelength is set by the type of refractometer, both environment and sample temperature can differ in between readings. Therefore, generally, temperature compensating scales are used in the commercial hand-held refractometers with an operation range between 10 and 35°C. Nevertheless, due to the above mentioned influencing factors, the effect of the environmental temperature (4°C-20°C-37°C), sample temperature (4°C-20°C-37°C) and number of freeze-thaw cycles (1–3) was evaluated on 20 giant panda urine samples with the Pal-USG (Cat) refractometer (USpG range: 1.002–1.020). The sample USpG was not influenced by the varying environmental or sample temperature, nor by repeated freeze-thaw cycles.

To make these refractometers more appealing for use in valuable, rare and frequently low-volume wildlife urine samples, the minimum required sample volume for reliable read-out was investigated on 30 giant panda urine samples. Hence, the advised 300 μL by the manufacturer sometimes still exceeds the available sample volume, especially since USpG is being measured as a correction factor prior to a panel of additional measurements for reproductive endocrine monitoring, e.g. based on immunoassays, requiring additional sample volume. In this subset of experiments, 10 volumes were challenged i.e. 300–250–200–150–100–90–80–70–60–50 μL. Reproducible outcomes could be measured starting form 100 μL, which was also the volume covering the complete ‘eye’ of the instrument. If the instrument’s monocle is properly cleaned in between samples, it is also possible to collect the sample back into a collection vial for later analysis.

In a study of Wyness et al. [32] a different model of handheld digital refractometers was challenged, reporting 4 decimals rather than 3. Wildlife endocrinologists should be encouraged to consider purchasing a similar model with the fourth decimal allowing a higher measurement accuracy, as well as, a multi-scale model in case application in other wildlife or domestic species will also be explored.

#### Creatinine

Five dilution factors were tested for creatinine analysis in seven reference samples of Hao Hao within a USpG range of 1.002 (most diluted) to 1.018 (most concentrated). For samples with a USpG value between 1.002 and 1.006, dilution factors of 2 to 5 were optimal for creatinine monitoring, whilst a factor of 20 to 40 yielded consistent results in more concentrated samples (USpG 1.009–1.018). Despite the predictive value of USpG for a sample’s dilution factor, the factor 40 was chosen for creatinine assessment in all monitored samples of Hao Hao. This dilution factor was optimal in samples with a high USpG, and suboptimal, but acceptable, in samples with a lower USpG. An overestimation of the creatinine concentration with less than 10% was observed in the latter samples.

### Comparison of the creatinine and USpG dataset

In this study, four urinary metabolites, previously described as relevant in reproductive monitoring of giant pandas, were analysed routinely to determine the optimal timing for successful insemination and follow-up of the potential gestation period in two female pandas (5 + 1 cycles). In contrast to any other comparable case study reported in literature, normalisation of raw urinary concentrations was performed based on USpG in comparison to the generally accepted creatinine-based correction.

The descriptives (mean, standard deviation, median and range) of all metabolites during the defined reproductive periods can be consulted in Tables 2 and 3 (and S1–S5 Tables).

**Table 2:** Descriptives for Tian Tian’s (SB569) reproductive cycle: USpG-, creatinine-corrected and raw metabolite concentration, USpG-values and creatinine concentrations in urine, faecal output and bodyweight.

	Anoestrus (min-max: D-208-D-9)		Pro-oestrus (min-max: D-14-D0)		Postoestrus (min-max:D1-D7)		Primary P4 rise (min-max:D8-D128)		Secondary P4 rise (min-max: D98-D179)	
	Mean (stdev)	Median (range)	Mean (stdev)	Median (range)	Mean (stdev)	Median (range)	Mean (stdev)	Median (range)	Mean (stdev)	Median (range)
**Oestrogens**	**(n = 449/526)**		**(n = 92/123)**		**(n = 26/50)**		**(n = 447/525)**		**(n = 203/247)**	
USpG (ng/mL)	1.44 (0.54)	1.37 (0.55–4.58)^a^	30.13 (21.10)	27.86 (2.13–86.93)^c^	11.80 (18.66)	2.35 (0.31–65.12)^b^	1.05 (0.40)	0.99 (0.35–3.21)^a^	1.58 (0.70)	1.50 (0.22–4.20)^a^
Creatinine (ng/mg Cr)	2.11 (1.07)	1.87 (0.66–13.64)^a^	14.31 (7.97)	14.05 (2.33–31.20)^c^	7.79 (8.70)	2.75 (0.28–27.49)^b^	1.77 (0.89)	1.60 (0.52–10.31)^a^	1.61 (1.02)	1.40 (0.25–7.98)^a^
Raw (ng/ mL)	1.72 (2.06)	1.17 (0.06–34.11)^a^	87.62 (80.92)	65.78 (0.43–350.1)^c^	44.95 (68.59)	6.51 (0.51–244.2)^b^	0.93 (0.60)	0.78 (0.14–4.43)^a^	2.39 (1.92)	1.92 (0.14–12.59)^a^
**Progesterone**	**(n = 176/526)**		**(n = 35/123)**		**(n = 14/50)**		**(n = 347/525)**		**(n = 169/247)**	
USpG (ng/mL)	5.24 (1.87)	4.9 (1.39–14.08)^a^	3.04 (1.01)	2.79 (1.81–5.80)^a^	5.01 (2.09)	4.56 (1.95–8.29)^a^	13.89 (6.10)	12.56 (5.14–47.95)^a^	62.73 (40.33)	54.35 (3.26–280.5)^b^
Creatinine (ng/mg Cr)	8.40 (3.59)	7.29 (2.89–24.38)^a^	2.07 (1.38)	1.63 (0.93–9.08)^a^	4.15 (2.81)	3.20 (1.11–11.05)^a^	25.36 (16.73)	20.05 (4.88–152.4)^b^	65.19 (56.70)	55.23 (1.82–578.9)^c^
Raw (ng/ mL)	5.04 (3.26)	4.19 (0.57–24.60)^a^	7.33 (3.79)	6.76 (1.21–16.89)^a^	12.66 (8.39)	11.89 (0.87–35.00)^a^	11.21 (5.89)	9.83 (2.13–38.14)^a^	89.37 (77.63)	75.86 (0.46–436.3)^b^
**Ceruloplasmin**	**(n = 174/526)**		**(n = 37/123)**		**(n = 12/50)**		**(n = 418/525)**		**(n = 183/247)**	
USpG (ng/mL)	9.84 (13.10)	5.00 (0.05–90.60)^a^	11.29 (8.35)	12.08 (0.08–30.31)^a^	17.22 (11.32)	9.76 (6.33–30.70)^a^^,^^b^	33.08 (26.27)	27.36 (0.05–156.1)^b^	10.21 (13.38)	6.00 (0.00–82.00)^a^
Creatinine (ng/mg Cr)	15.24 (24.87)	6.06 (0.07–189.38)^a^	6.24 (4.62)	5.81 (0.04–15.54)^a^	22.59 (19.89)	14.91 (2.44–71.29)^a^	64.97 (84.20)	48.69 (0.06–1319)^b^	17.33 (36.11)	5.82 (0.00–255.3)^a^
Raw (ng/ mL)	10.85 (12.99)	5.00 (0.00–69.20)^a^	28.85 (20.90)	29.10 (0.10–78.00)^b^^,^^c^	34.54 (26.91)	25.60 (5.57–108.9)^c^	24.44 (18.44)	21.80 (0.00–136.6)^b^	12.25 (12.05)	7.65 (0.00–57.02)^a^
**PGFM**	**(n = 10/526)**		**(n = 32/123)**		**(n = 11/50)**		**(n = 157/525)**		**(n = 189/247)**	
USpG (ng/mL)	10.87 (5.47)	9.68 (3.72–22.30)^a^^,^^b^	28.81 (17.19)	22.63 (7.79–83.79)^a^^,^^b^	25.57 (28.73)	16.38 (3.96–101.8)^a^^,^^b^	5.67 (3.40)	4.60 (0.0–16.41)^a^	36.75 (45.19)	15.1 (1.89–308.1)^b^
Creatinine (ng/mg Cr)	14.07 (8.26)	11.68 (5.02–32.87)^a^	14.93 (6.05)	13.07 (7.82–32.13)^a^	13.40 (9.88)	10.53 (5.53–41.75)^a^	10.49 (8.28)	7.80 (1.91–52.38)^a^	29.96 (50.01)	15.79 (2.31–536.49)^a^
Raw (ng/ mL)	19.40 (17.55)	14.53 (3.25–61.33)^a^^,^^b^	78.81 (60.85)	62.58 (13.63–261.8)^c^	54.44 (96.48)	21.10 (0.86–381.7)^a^^,^^b^^,^^c^	4.67 (4.54)	3.29 (0.81–39.0)^a^	58.66 (75.01)	20.81 (0.00–318.3)^b^^,^^c^
**USpG**	**(n = 497/526)**		**(n = 94/123)**		**(n = 31/50)**		**(n = 509/525)**		**(n = 221/247)**	
USpG	1.009 (0.006)	1.007 (1.000–1.034)^a^	1.021 (0.007)	1.023 (1.002–1.037)^c^	1.019 (1.011)	1.022 (1.000–1.033)^c^	1.007 (0.004)	1.006 (1.000–1.024)^a^	1.012 (0.008)	1.011 (1.001–1.042)^b^
**Cr**	**(n = 525/526)**		**(n = 123/123)**		**(n = 48/50)**		**(n = 518/525)**		**(n = 244/247)**	
Creatinine (mg/mL)	0.87 (0.75)	0.66 (0.00–4.64)^a^	5.33 (3.14)	4.92 (0.00–13.23)^d^	4.11 (2.76)	4.09 (0.03–10.13)^c^	0.56 (0.45)	0.47 (0.00–3.50)^a^	1.91 (2.17)	1.27 (0.00–13.42)^b^
**Faeces**	**(n = 451/526)**		**(n = 37/123)**		**(n = 17/50)**		**(n = 451/525)**		**(n = 193/247)**	
Faeces (kg)	3.72 (1.42)	3.70 (0.90–7.50)^b^	1.43 (0.79)	1.30 (0.20–3.90)^a^	2.56 (1.65)	1.60 (1.00–5.70)^a^^,^^b^	8.66 (2.16)	8.80 (1.60–15.90)^c^	3.70 (3.18)	2.50 (0.20–13.80)^b^
**Bodyweight**	**(n = 437/526)**		**(n = 16/123)**		**(n = 9/50)**		**(n = 448/525)**		**(n = 84/247)**	
Bodyweight (kg)	107.5 (2.04)	107.5 (102.0–112.0)^b^	103.9 (2.01)	104.1 (99.7–107.0)^a^^,^^b^	101.2 (2.04)	102.0 (98.5–104.0)^a^	111.9 (5.6)	112.2 (100.6–126.5)^c^	114.9 (4.4)	115.5 (105.0–123.4)^c^

Stdev = standard deviation; n = number of samples; USpG = urinary specific gravity; cr = creatinine.

^a-d^ = different superscripts (^a-d^; ascending; horizontally) indicate significant differences for the respective metabolite levels between each defined reproductive period; Independent-Samples Kruskall Wallis test with post hoc Dunn’s comparison; significant if p < 0.05.

**Table 3:** Descriptives for Hao Hao’s (SB741) 2016 reproductive cycle: USpG-, creatinine-corrected and raw metabolite concentration, USpG-values and creatinine concentrations in urine and faecal output.

	Pro-oestrus (D-8 -D0)		Postoestrus (D0—D6)		Primary P4 rise (D7—D76)		Secondary P4 rise (D77-D114)		Post Birth (D119-D125)	
	Mean (stdev)	Median (range)	Mean (stdev)	Median (range)	Mean (stdev)	Median (range)	Mean (stdev)	Median (range)	Mean (stdev)	Median (range)
**Oestrogens**	**(n = 28/28)**		**(n = 17/17)**		**(n = 64/65)**		**(n = 49/49)**		**(n = 5/5)**	
USpG (ng/mL)	83.41 (61.31)	63.03 (31.61–306.5)^b^	27.46 (24.87)	16.86 (2.62–88.82)^a^	2.67 (1.19)	2.42 (0.85–6.80)^a^	6.61 (3.62)	5.82 (1.31–14.89)^a^	2.43 (1.86)	1.66 (0.50–5.29)^a^
Creatinine (ng/mg Cr)	94.60 (55.13)	80.71 (36.36–249.1)^c^	41.83 (47.51)	20.36 (3.57–189.7)^b^	12.87 (33.76)	6.63 (2.10–272.7)^a^^,^^b^	8.07 (4.60)	7.32 (2.88–30.46)^a^^,^^b^	2.10 (0.81)	1.84 (1.12–3.18)^a^
Raw (ng/ mL)	84.18 (71.06)	75.49 (16.33–391.0)^b^	31.67 (26.96)	21.48 (1.56–77.49)^a^	1.99 (1.01)	1.85 (0.46–4.82)^a^	10.07 (8.71)	6.72 (0.76–35.46)^a^	6.89 (6.93)	4.49 (0.54–18.14)^a^
**Progesterone**	**(n = 28/28)**		**(n = 17/17)**		**(n = 64/65)**		**(n = 49/49)**		**(n = 5/5)**	
USpG (ng/mL)	3.00 (1.22)	2.60 (1.63–7.27)^a^	6.85 (2.78)	5.90 (3.40–13.27)^a^	14.39 (6.28)	13.90 (5.69–37.54)^a^	87.30 (43.85)	86.40 (26.60–174.9)^b^	6.22 (2.89)	5.21 (3.80–10.99)^a^
Creatinine (ng/mg Cr)	3.48 (1.04)	3.21 (2.10–5.75)^a^	12.67 (15.20)	7.32 (4.94–67.26)^a^	62.63 (127.5)	32.43 (3.54–999.8)^a^^,^^b^	129.83 (88.75)	117.61 (19.87–372.9)^b^	6.27 (1.40)	5.76 (4.83–8.51)^a^
Raw (ng/ mL)	2.95 (1.30)	2.83 (0.71–6.81)^a^	8.88 (7.02)	6.12 (0.61–29.00)^a^	9.77 (4.61)	8.58 (0.92–18.94)^a^	112.16 (74.17)	93.30 (14.39–334.6)^b^	16.26 (13.01)	11.77 (4.11–37.64)^a^
**Ceruloplasmin**	**(n = 28/28)**		**(n = 17/17)**		**(n = 64/65)**		**(n = 49/49)**		**(n = 5/5)**	
USpG (ng/mL)	6.50 (3.51)	5.44 (1.73–15.55)^b^^,^^c^	4.49 (2.62)	4.69 (0.73–8.79)^a^^,^^b^	10.18 (3.98)	9.96 (2.44–19.18)^c^	4.60 (3.49)	3.63 (0.61–18.01)^a^^,^^b^	0.71 (0.30)	0.82 (0.19–0.91)^a^
Creatinine (ng/mg Cr)	7.51 (3.97)	6.87 (2.24–22.22)^a^^,^^b^	12.72 (2.62)	6.84 (0.47–98.47)^a^^,^^b^	35.26 (35.21)	29.19 (9.07–280.0)^b^	10.45 (14.89)	3.96 (0.49–56.72)^a^^,^^b^	0.85 (0.70)	0.68 (0.21–2.04)^a^
Raw (ng/ mL)	6.28 (3.50)	5.49 (2.03–14.02)^b^	4.75 (3.16)	3.23 (0.90–11.34)^a^^,^^b^	7.69 (4.32)	6.41 (2.73–20.42)^b^	4.59 (2.33)	4.34 (1.20–12.47)^a^^,^^b^	1.71 (1.06)	1.93 (0.35–3.06)^a^
**PGFM**	**(n = 28/28)**		**(n = 17/17)**		**(n = 29/65)**		**(n = 49/49)**		**(n = 5/5)**	
USpG (ng/mL)	14.03 (8.52)	12.38 (2.51–34.82)^a^	15.52 (11.34)	14.47 (3.10–52.40)^a^	2.75 (1.02)	2.72 (1.21–6.34)^a^	119.58 (254.71)	26.64 (2.07–1344)^a^	40.76 (25.98)	33.37 (9.52–76.92)^a^
Creatinine (ng/mg Cr)	17.54 (12.38)	13.44 (3.71–46.06)^a^	24.03 (17.73)	18.66 (7.07–66.55)^a^	10.02 (8.43)	7.11 (2.98–45.12)^a^	92.87 (171.48)	24.96 (5.99–857.3)^a^	36.34 (10.68)	36.87 (20.77–46.16)^a^
Raw (ng/ mL)	13.70 (9.30)	11.43 (2.71–39.95)^a^	24.50 (34.69)	19.18 (0.61–151.2)^a^	2.12 (1.26)	1.70 (0.81–5.61)^a^	281.53 (767.56)	38.61 (1.23–4605)^a^	112.18 (98.34)	77.35 (10.03–263.6)^a^
**USpG**	**(n = 28/28)**		**(n = 17/17)**		**(n = 65/65)**		**(n = 49/49)**		**(n = 5/5)**	
USpG	1.006 (0.003)	1.006 (1.002–1.011)^a^^,^^b^	1.007 (0.004)	1.007 (1.001–1.016)^b^	1.004 (0.002)	1.004 (1.002–1.012)^a^	1.007 (0.004)	1.007 (1.002–1.019)^b^	1.013 (0.005)	1.015 (1.006–1.019)
**Cr**	**(n = 28/28)**		**(n = 17/17)**		**(n = 64/65)**		**(n = 49/49)**		**(n = 5/5)**	
Creatinine (mg/mL)	0.90 (0.44)	0.85 (0.26–1.92)^a^^,^^b^	1.19 (1.10)	0.92 (0.09–4.24)^b^^,^^c^	0.29 (0.18)	0.26 (0.01–0.82)^a^	1.52 (1.34)	1.07 (0.09–5.37)^c^	2.70 (1.97)	2.44 (0.48–5.71)
**Faeces**	**(n = 6/28)**		**(n = 4/17)**		**(n = 61/65)**		**(n = 44/49)**			
Faeces (kg)	4.97 (1.09)	4.85 (3.70–6.60)^a^	8.53 (2.48)	9.30 (5.00–10.50)^a^^,^^b^	12.64 (4.31)	14.00 (3.70–22.00)^b^	5.47 (6.38)	2.80 (1.00–23.20)^a^		

Stdev = standard deviation; n = number of samples; USpG = urinary specific gravity; cr = creatinine.

^a-c^ = different superscripts (^a-c^; ascending; horizontally) indicate significant differences for the respective metabolite levels between each defined reproductive period; Independent-Samples Kruskall Wallis test with post hoc Dunn’s comparison; significant if p < 0.05.

#### Biological aspects

When metabolite profiles corrected for USpG are pairwise compared with the profiles corrected for creatinine, significant differences in levels and profiles are predominantly observed during pro-oestrus and primary progesterone rise (Table 4). This discrepancy is potentially largely resulting from the interaction of physiological features on each of these normalisation factors. Hormonal changes induce altered behaviour, including increased or decreased appetite. In this study, the latter was monitored by recording daily faecal output and bodyweight, which are clearly correlated (r = 0.629–0.782) (Table 5). In this study, the data-collections of faecal output and bodyweights have been shown extremely relevant, not only in explaining the main results of this study, but also in supporting the interpretation of the reproductive endocrine profile, allowing the prediction of upcoming changes in the reproductive metabolome. Since faecal output has a shorter lag period compared to bodyweight (less than one day), can be monitored without specialized equipment, is completely non-invasive and not requiring any cooperation of the animal, and with removal of faecals being part of the daily routine, it should be strongly encouraged to record these data throughout the cycle, especially when daily weighing of the panda becomes challenging or clearly shows to be stress-inducing.

**Table 4:** Comparison of the USpG corrected dataset with the Cr corrected dataset for each monitored metabolite in each observed cycle.

	Anoestrus		Pro-oestrus		Postoestrus		Primary P4 rise		Secondary P4 rise		Post Birth	
	n	p-value	n	p-value	n	p-value	n	p-value	n	p-value	n	p-value
**Oestrogens**												
Tian Tian 2013	60	0.000	46	0.000	12	0.002	74	0.000	51	0.500		
Tian Tian 2014	64	0.000	9	0.008	3	0.593	77	0.000	56	0.304		
Tian Tian 2015	75	0.000	15	0.005	4	0.465	100	0.000	19	0.355		
Tian Tian 2016	167	0.000	11	0.008	1	0.317	99	0.000	37	0.108		
Tian Tian 2017	83	0.000	11	0.008	6	0.116	97	0.000	40	0.040		
Hao Hao 2016			28	0.005	17	0.035	64	0.000	49	0.171	5	0.893
**Progesterone**												
Tian Tian 2013					1	0.317	31	0.000	22	0.498		
Tian Tian 2014	21	0.000	9	0.008	3	0.593	77	0.000	56	0.063		
Tian Tian 2015	75	0.000	15	0.006	4	1.000	100	0.000	19	0.629		
Tian Tian 2016							42	0.000	32	0.036		
Tian Tian 2017	80	0.000	11	0.008	6	0.116	97	0.000	40	0.008		
Hao Hao 2016			28	0.006	17	0.124	64	0.000	49	0.013	5	0.893
**Ceruloplasmin**												
Tian Tian 2013			1	0.317	1	0.317	31	0.000	30	0.974		
Tian Tian 2014					1	0.317	77	0.000	56	0.068		
Tian Tian 2015	32	0.000	15	0.027	4	0.715	96	0.000	19	0.243		
Tian Tian 2016	125	0.000	11	0.013	1	0.317	100	0.000	38	0.122		
Tian Tian 2017	17	0.001	10	0.013	5	0.500	114	0.000	40	0.058		
Hao Hao 2016			28	0.011	17	0.035	64	0.000	49	0.032	5	0.893
**PGFM**												
Tian Tian 2013							16	0.001	38	0.149		
Tian Tian 2014							19	0.000	56	0.649		
Tian Tian 2015			12	0.005	4	0.465	55	0.000	18	0.845		
Tian Tian 2016	4	0.465	11	0.010	1	0.317	28	0.000	37	0.001		
Tian Tian 2017	6	0.028	9	0.021	6	0.075	39	0.000	40	0.001		
Hao Hao 2016			28	0.002	17	0.028	29	0.000	49	0.717	5	0.893

Reported concentrations were significant different if p < 0.05 (non-parametric paired comparison with the Wilcoxon Signed Rank test).

USpG = urinary specific gravity; cr = creatinine; n = number of samples.

**Table 5:** Pearson’s correlation coefficients for USpG, creatinine, faecal output and bodyweight for each complete cycle or for the respective periods within each cycle.

	Anoestrus		Pro-oestrus		Postoestrus		Primary P4 rise		Secondary P4 rise		Complete cycle	
	n	Pearson’s r	n	Pearson’s r	n	Pearson’s r	n	Pearson’s r	n	Pearson’s r	n	Pearson’s r
**USpG—Creatinine**												
Tian Tian 2013	60	0.940**	46	0.839**	12	0.388	78	0.731**	53	0.923**	249	0.823**
Tian Tian 2014	79	0.933**	10	0.984**	4	0.917	81	0.855**	58	0.873**	232	0.859**
Tian Tian 2015	76	0.931**	16	0.813**	5	0.975*	100	0.882**	20	0.789**	217	0.819**
Tian Tian 2016	190	0.915**	11	0.880**	4	0.884	120	0.903**	41	0.878**	366	0.805**
Tian Tian 2017	92	0.839**	11	0.735*	6	0.902*	125	0.872**	46	0.904**	280	0.894**
Hao Hao 2016			28	0.861**	17	0.805**	64	0.788**	49	0.969**	163	0.894**
**USpG—Faeces output**												
Tian Tian 2013	35	-0.392*	11	0.267	2	-1.000**	65	-0.004	44	-0.337*	157	-0.462**
Tian Tian 2014	78	-0.016					74	-0.168	39	-0.412*	182	-0.121
Tian Tian 2015	75	-0.024	7	0.128	3	-0.282	93	-0.149	22	-0.646**	200	-0.273**
Tian Tian 2016	159	-0.049	6	-0.339	3	-0.596	90	-0.378**	34	-0.405*	291	-0.338**
Tian Tian 2017	80	-0.077	6	0.176	3	0.327	116	0.275**	35	-0.632**	240	-0.535**
Hao Hao 2016			6	-0.126	4	-0.631	61	0.066	44	-0.539**	115	-0.497**
**Creatinine—Faeces output**												
Tian Tian 2013	47	-0.450**	14	0.139	5	0.079	70	-0.333**	56	-0.492**	192	-0.512**
Tian Tian 2014	86	-0.148					78	-0.190	39	-0.412*	207	-0.245**
Tian Tian 2015	76	-0.085	8	-0.567	4	-0.205	91	-0.198	20	-0.699**	199	-0.401**
Tian Tian 2016	161	-0.131	7	-0.394	3	-0.890	92	-0.454**	36	-0.494**	299	-0.383**
Tian Tian 2017	80	-0.189	6	-0.798	3	-0.826	116	-0.410**	35	-0.605**	240	-0.511**
Hao Hao 2016			6	-0.417	4	-0.934	61	0.097	44	-0.593**	115	-0.625**
**Bodyweight—Faeces output**												
Tian Tian 2013	48	0.143	6	0.420	5	0.776	70	0.705**	23	0.842**	152	0.678**
Tian Tian 2014	86	0.203					77	0.657**	21	0.795**	187	0.643**
Tian Tian 2015	75	0.390**	4	0.658			95	0.756**	16	0.824**	191	0.629**
Tian Tian 2016	144	0.526**					88	0.694**	9	0.613	242	0.718**
Tian Tian 2017	78	0.406**					114	0.721**			196	0.782**
**Bodyweight—Creatinine**												
Tian Tian 2013	47	0.116	6	-0.566	5	0.030	70	-0.508**	23	0.691**	151	-0.455**
Tian Tian 2014	86	0.143					78	0.393**	22	-0.754**	190	-0.144*
Tian Tian 2015	76	-0.085	6	0.496			92	-0.173	14	-0.785**	189	-0.140
Tian Tian 2016	147	-0.069					90	-0.459**	9	-0.911**	247	-0.382**
Tian Tian 2017	80	0.009					114	-0.348**			198	-0.350**
**Bodyweight—USpG**												
Tian Tian 2013	35	0.073	5	-0.511	2	1.000**	65	0.085	18	-0.562*	125	-0.252**
Tian Tian 2014	78	0.243*					74	0.412**	15	-0.582*	168	-0.015
Tian Tian 2015	75	-0.036	6	0.270			94	-0.167	16	-0.264	192	-0.158*
Tian Tian 2016	145	-0.008					88	-0.436**	9	-0.872**	243	-0.359**
Tian Tian 2017	80	0.053					114	-0.296**			198	-0.376**

Significant coefficients are flagged (significant if p < 0.05 (*); ** = p < 0.01).

USpG = urinary specific gravity; n = number of samples; r = correlation coefficient.

**Fig 1:**
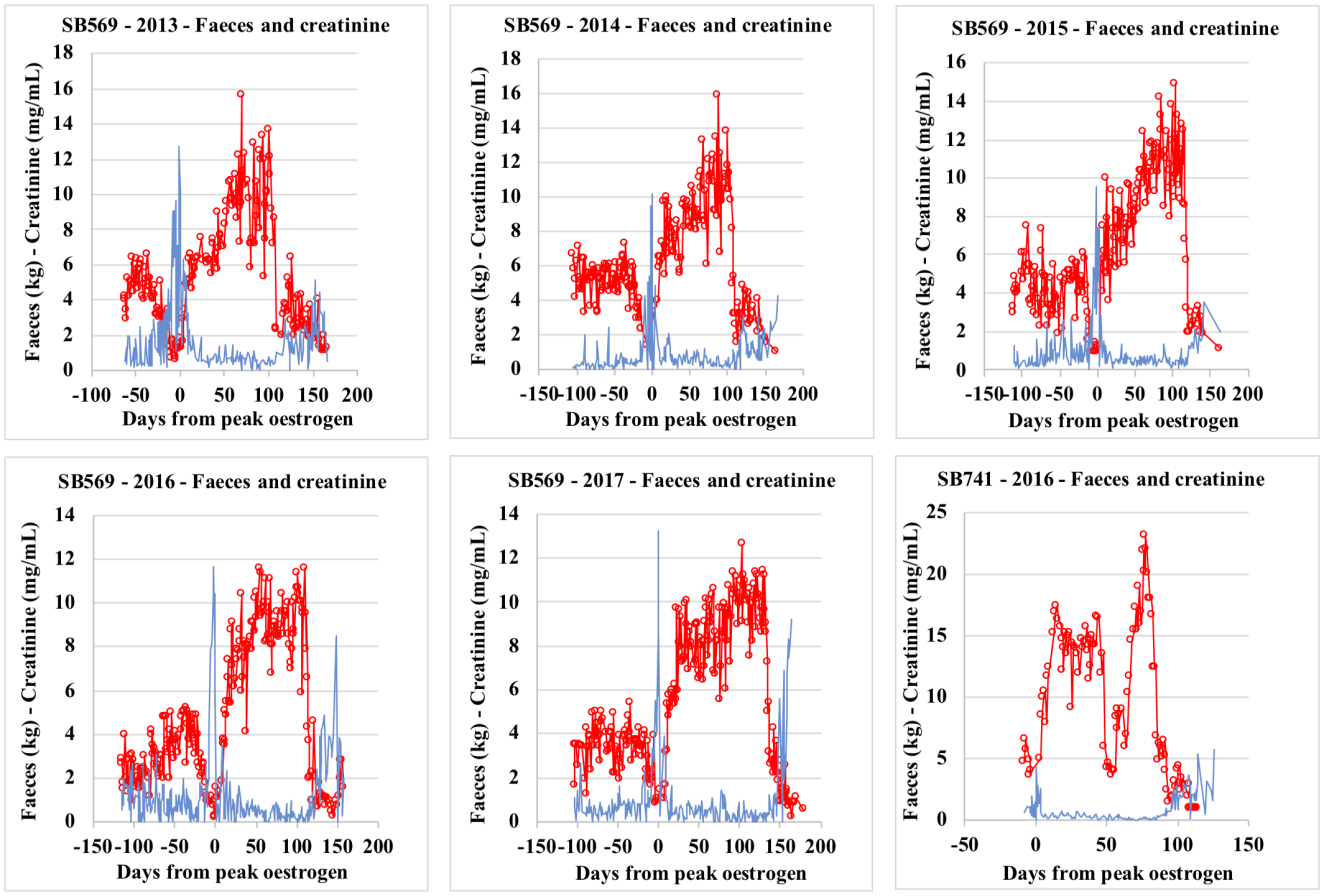
Faeces and creatinine profiles for Tian Tian_SB569_ (2013–2017) and Hao Hao_SB 741_(2016).

**Fig 2:**
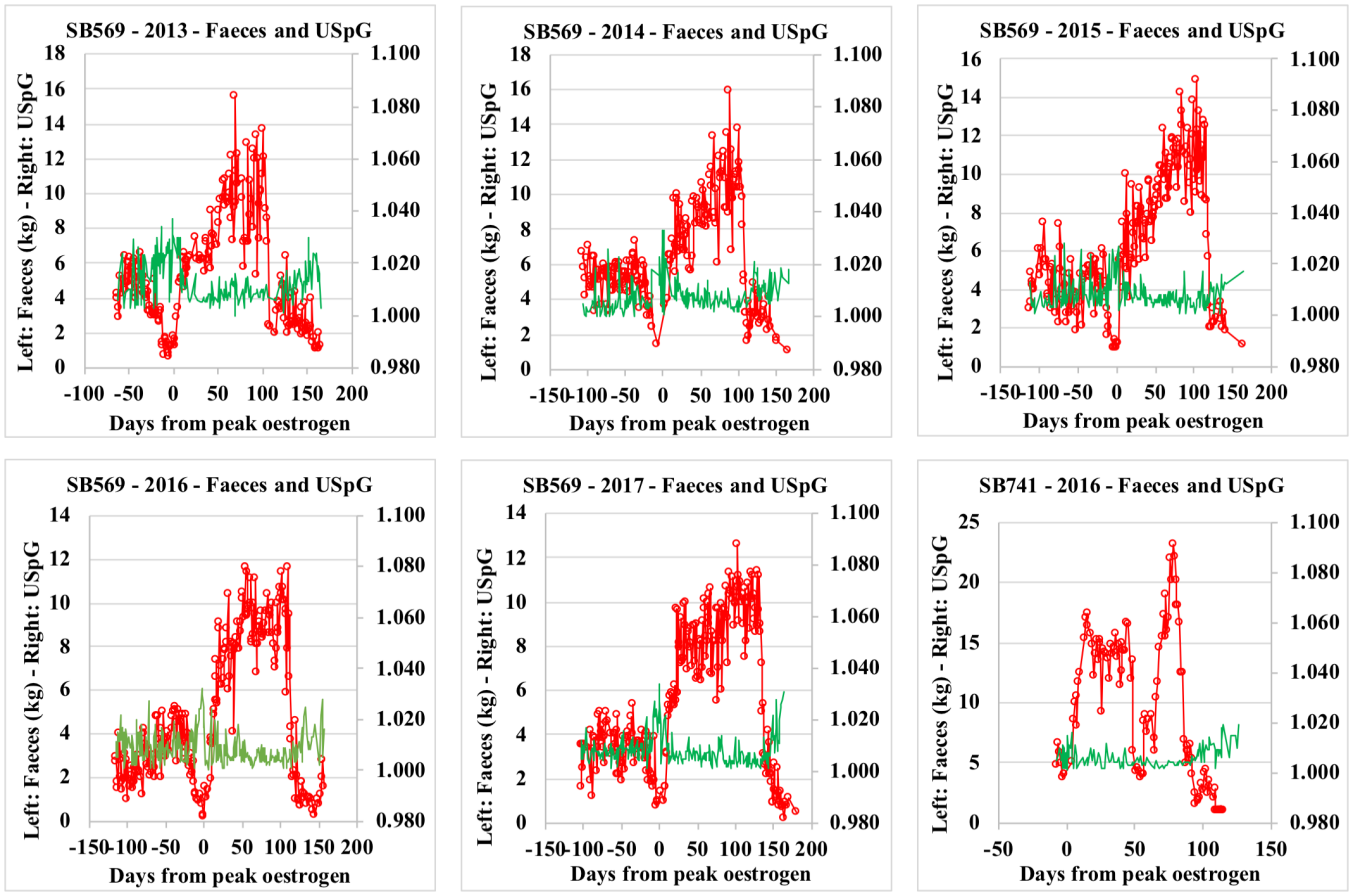
Faeces and urinary specific gravity (USpG) profiles for Tian Tian_SB569_ (2013–2017) and Hao Hao_SB 741_(2016).

In this study, lowest faecal output and bodyweight records were observed during oestrus, parallel to the highest creatinine and USpG values observed during the reproductive cycle. On the other hand, during the late primary progesterone rise, lowest creatinine and USpG values match a period of significant increase in faecal output and bodyweight (Tables 2 and 3 and S1–S5 Tables; Figs 1 and 2).

While no significant correlations between appetite-parameters and correction factors could be reported specifically during oestrus, a generally stronger negative correlation was demonstrated for creatinine compared to USpG in other reproductive periods and in the complete cycle, indicating creatinine being more influenced by these parameters than USpG (Table 5).

**Fig 3:**
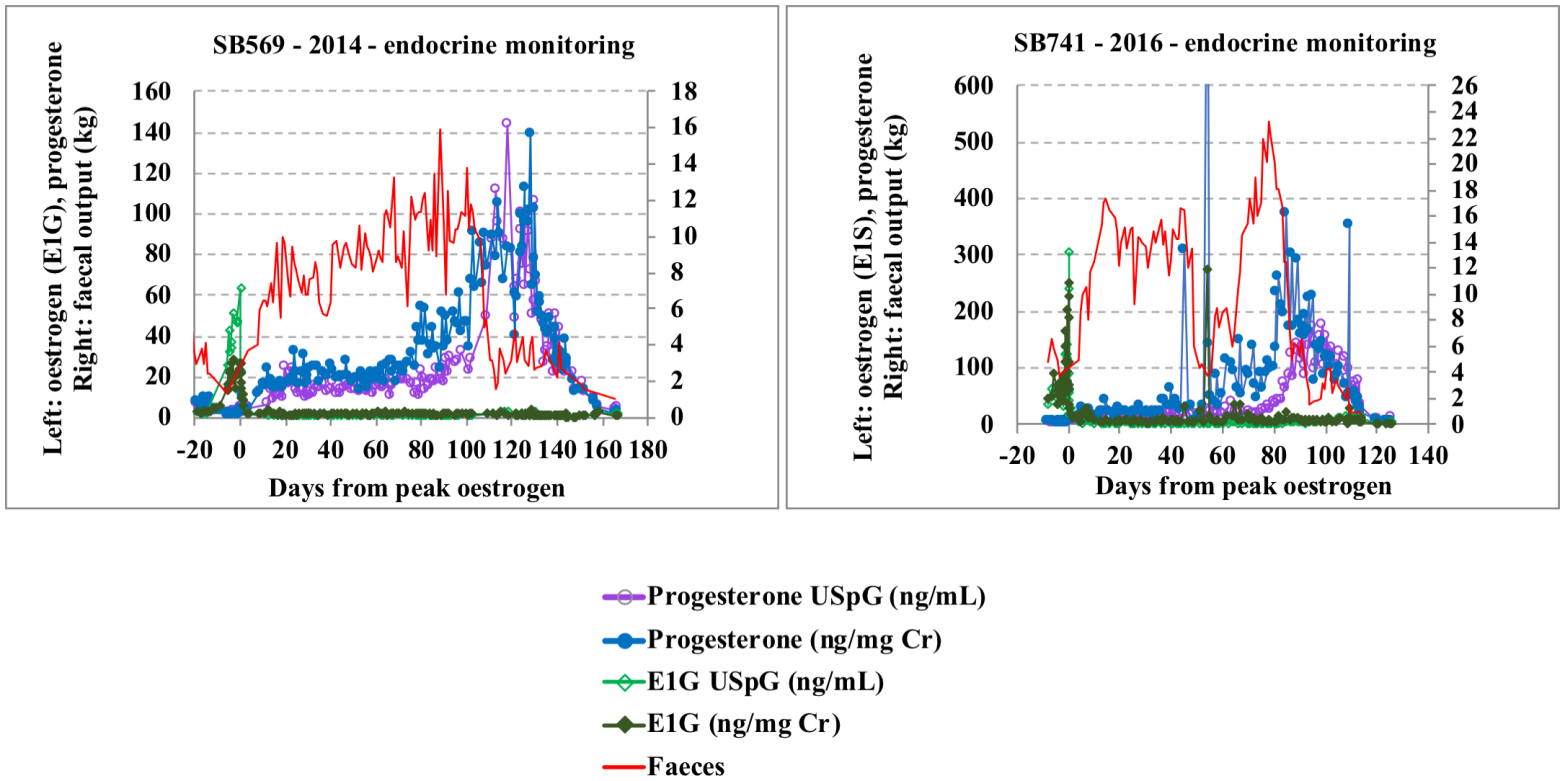
Faeces, oestrogen and progesterone full-cycle profiles (normalised with creatinine and USpG) for Tian Tian_SB569_ (2014) and Hao Hao_SB 741_(2016).

The above findings are in agreement with the literature, as it is well documented that creatinine excretion is complicated by diet- and exercise-related factors [22, 24, 25, 29, 35]. The giant pandas in our study clearly showed decreased appetite (including water intake) and increased activity during oestrus, likely responsible for increased creatinine, not solely as a result of dehydration, but additionally from increased muscle tissue breakdown. Conversely, an increased appetite together with a decreasing activity rate may be responsible for the low creatinine levels monitored during late primary rise (Fig 1, Tables 2 and 3 and S1–S5 Tables). More specifically, when observing Hao Hao’s pregnancy, creatinine values dropped below 0.1 mg/mL two weeks prior to secondary progesterone rise (= D65) with approximately 40% of the values below this threshold until one week into secondary progesterone rise (= D 85), and then remain low (below 0.5 mg/mL) for a further week (= D91-92). This 0.1 mg/mL Cr threshold is accepted as the lower limit for reliable correction in giant panda urine, leading to overestimation of the metabolite concentrations if values below this are being used. Re-establishment of the creatinine values occurred together with the increasing PGFM and progesterone values. At this time appetite is gradually reduced resulting in increasing creatinine concentrations towards the end of the secondary progesterone rise (Fig 3 and S1 Fig).

Other variables recently identified to influence creatinine levels–merely studied in humans–are inter- and intra-subject variation including the moment of sampling (time of day), the age, the gender and racial factors [35, 36]. Diet related factors can also affect USpG but with less impact compared to creatinine [22, 29, 35]. The abrupt changes in appetite in these female pandas also considerably impact the total range observed between minimum and maximum values of USpG, however USpG remains within acceptable margins (Fig 2). USpG is clearly less affected than creatinine, with USpG mainly indirectly altered by changes in hydration status and potentially also dietary composition. Creatinine on the other hand is directly influenced by increasing concentrations of reproductive hormones affecting activity rate and tissue turnover as well as nutritional intake. As a result, the use of creatinine as a correction factor may lead to falsely high (low creatine) or low (high creatine) hormone levels, as demonstrated in this study.

For the periods where endocrine changes clearly trigger changes in appetite, the discrepant impact on both normalisation factors is further demonstrated by the decreased correlation between the USpG and creatinine, with generally a lower correlation observed during pro-oestrus and primary progesterone rise (Table 5). This is further emphasized by the significantly different levels observed in these periods when evaluating the USpG and creatinine corrected dataset for each metabolite (Tables 2, 3 and 4, S1–S5 Tables).

**Fig 4:**
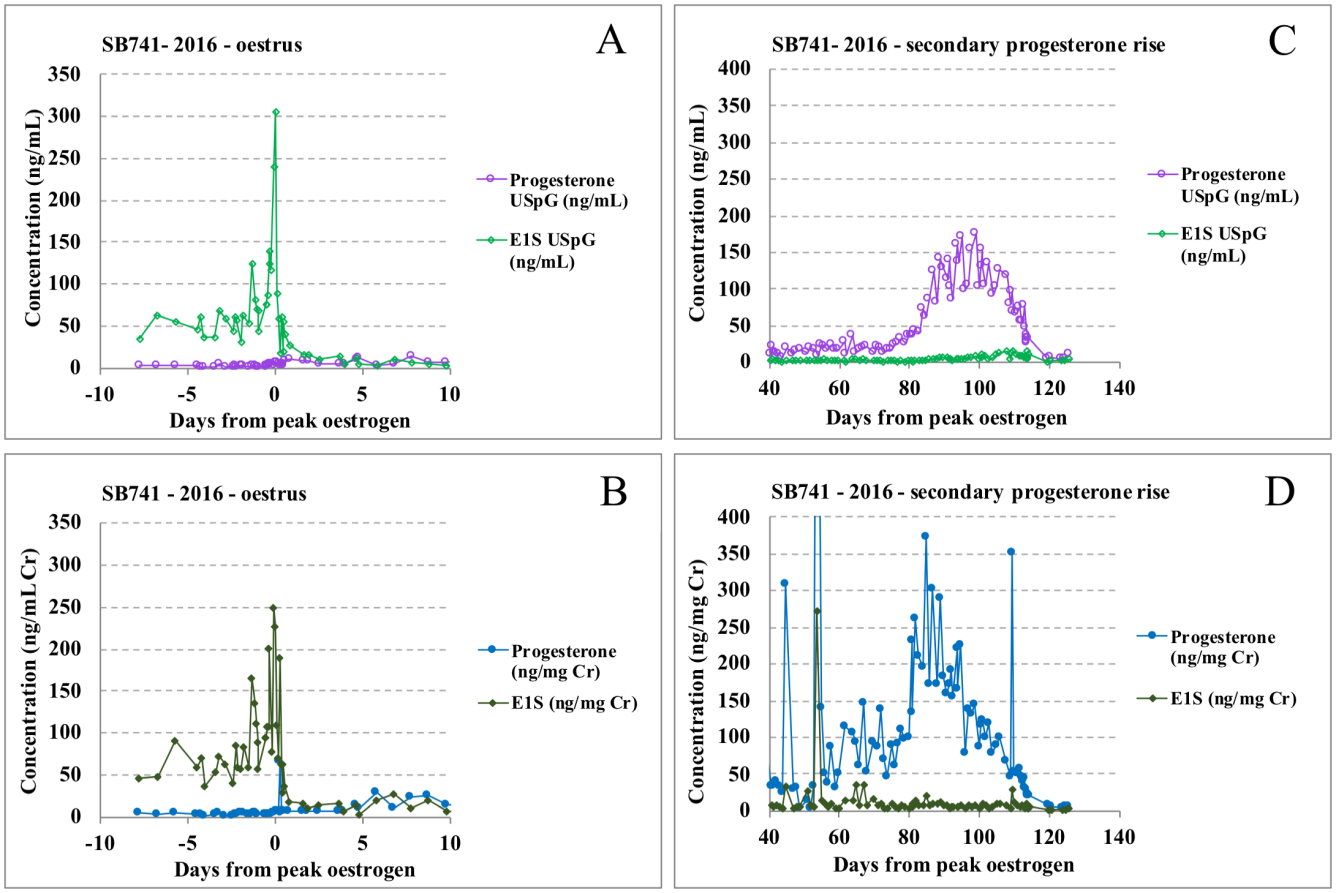
Oestrus profiles for Hao Hao_SB741_. Profiles corrected with USpG (A) and creatinine (B): a sharper and significant oestrogen peak without confounding same-level pre-and post- peak peaks demonstrates the superiority of USpG normalisation in breeding management. Progesterone profiles for Hao Hao corrected with USpG (C) and creatinine (D): the irregular skewed progesterone profile with outliers and a two-week earlier onset of secondary rise demonstrates the difficulty in interpreting creatinine-normalised profiles.

**Fig 5:**
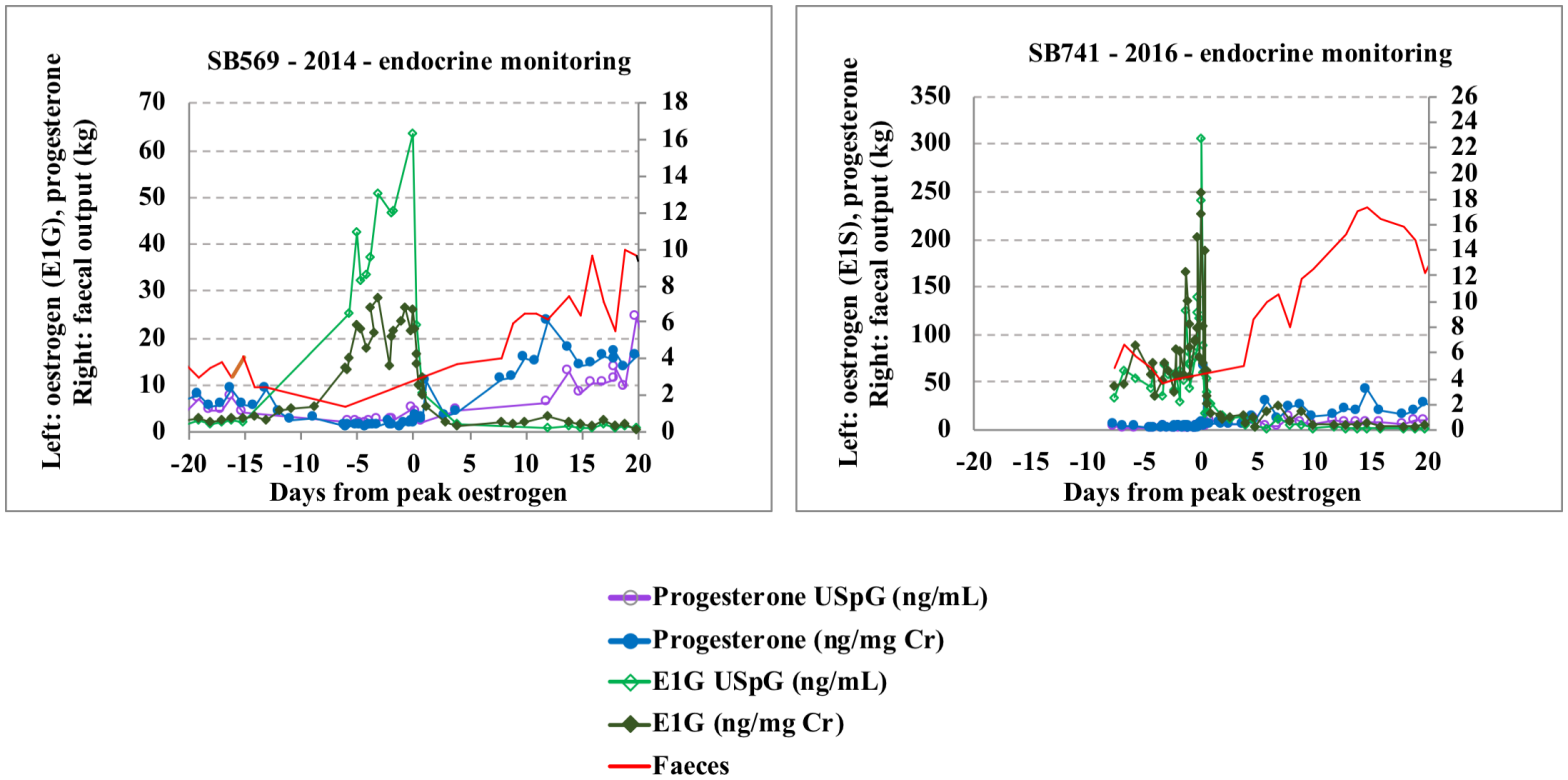
Faeces, oestrogen and progesterone oestrus profiles (normalised with creatinine and USpG) for Tian Tian_SB569_ (2014) and Hao Hao_SB 741_(2016).

For all metabolites, concentrations are significantly higher during oestrus in USpG corrected profiles, while values during anoestrus and primary progesterone rise are lower compared to the creatinine corrected dataset (Table 4). Consequently, the USpG profile renders a lower baseline and thus a more distinct increasing oestrogen profile during pro-oestrus followed by a sharp oestrogen peak at oestrus with steep decline (Figs 4A, 4B and 5). In contrast, interpretation of the creatinine profile is hampered by higher baseline levels and by an unclear oestrogen peak which is preceded and followed by almost equally-high pre- and post- oestrogen peak values. As a result, the USpG profile, with a distinct oestrogen peak followed by an abrupt decline, allows better pinpointing of the most suitable timing for fertilization by natural mating or artificial insemination and will therefore also match better with observed behavioural changes (Figs 4A, 4B and 5 and S2 Fig).

Approximately two to three weeks prior to the start of the secondary progesterone rise, lowest creatinine values, additionally characterized by low day-to-day variability and co-occurring with increased faecal output, can be observed in all cycles until early secondary progesterone rise, at which point PGFM levels start to increase coincident with decreasing faecal output. As a result of these appetite-induced significant changes in creatinine, these corrected progesterone levels tend to falsely increase earlier and more inconsistently. In Figs 3, 4C and 4D the superior performance of USpG for progesterone normalisation during primary and secondary progesterone rise is clearly demonstrated, with the absence of outliers, a more distinct and delayed onset of secondary progesterone rise and a regular progesterone profile.

#### Technical aspects

Besides the more modest impact of the physiological changes related to oestrus and (pseudo)pregnancy on USpG compared to creatinine, the more consistent outcome and smoothening of the profile with USpG can also partly be attributed to the correction formulas behind each method for normalisation. The latter is the likely explanation for the significant higher baseline levels observed for all metabolites in the creatinine dataset during anoestrus (Tables 2 and 3 and S1–S5 Tables). Creatinine correction entails a simple ratio, and therefore the outcome will be hugely influenced by minor changes in the denominator. The USpG correction on the other hand, is performed by an equation taking into account the average population value, therefore balancing the outcomes and hence smoothing out the profiles.

The application of USpG as a novel tool for correction in wildlife species has a further noteworthy advantage compared to creatinine. Creatinine monitoring is usually performed based on colorimetric assays exploiting specific chemical reactions. These quantitative assays require specialized laboratory equipment and technical know-how from the applicator. Commercial kits can be costly and exact quantification can only be achieved based on a calibration curve (subject to limits of detection and quantification) after adequate sample dilution. In contrast, USpG can be monitored on the raw sample with an easily handled hand-sized refractometer not subjected to any limits of detection/quantification as the gravity of any urine sample will exceed that of distilled water. Refractometers are generally considered robust instruments and the long-term performance of an instrument can be easily monitored by periodical in-house calibration and (re)validation [37]. The instrument can be operated by an unexperienced person without any implications on the results. Additionally, this instrument does not require any other supporting equipment (e.g. an optical density reader) and is therefore suitable to be part of ambulatory laboratories, especially in field conditions. Moreover, it can be used in a wide variety of environmental conditions without major impacts on the results (operating temperature: 10–35°C), in contrast to any colorimetric assay of which the performance is usually prone to varying upon differing environmental conditions. USpG detection does not require any sample pre-treatment and may easily provide an indication on required sample dilution factors prior to the monitoring of metabolites of interest with enzymatic immunoassays. In general, it has an unlimited application for fast and easy monitoring. Correction with USpG may therefore offer new perspectives for those research groups avoiding metabolite monitoring in urine because of normalisation issues attributed to the potentially complicated and expensive creatinine measurements. Several research groups are indeed in favour of faecal metabolite monitoring; however, this generally includes elaborate faecal extractions whereby concentration outcomes may be influenced by the animals’ diet and overall food uptake [8, 38].

## Conclusion

In this study, the successful and easy application of USpG monitoring as an alternative tool for urinary metabolite concentration correction in the giant panda was incontestably demonstrated. While creatinine and USpG were both equally performing in correcting for an animal’s hydration status, a smoother and more reliable profile, particularly during oestrus and late primary progesterone rise was clearly observed in the USpG dataset. The latter may assist in optimizing mating or insemination strategies and fine-tuning the definition of the *corpus luteum* dormancy phase and active luteal phase, as such providing an added value for any study unravelling the metabolic profile of reproductive features in the giant panda, more specifically elucidating exact timings of implantation and pregnancy duration.

Undoubtedly, USpG analysis will have similar practical and scientific advantages in other wildlife species and the exploration of its future application in any of these species should therefore be strongly encouraged.

## Supporting information

S1 Fig Faeces, oestrogen and progesterone oestrus profiles (normalised with creatinine and USpG) for Tian Tian_SB569_ (2013, 2015–2017). (TIF)

S2 Fig Faeces, oestrogen and progesterone full-cycle profiles (normalised with creatinine and USpG) for Tian Tian_SB569_ (2013, 2015–2017). (TIF)

S1 Table Descriptives for Tian Tian’s 2013 reproductive cycle (SB569): USpG-, creatinine-corrected and raw metabolite concentration, USpG-values and creatinine concentrations in urine, faecal output and bodyweight. Stdev = standard deviation; n = number of samples; USpG = urinary specific gravity; cr = creatinine. Different superscripts (a-d; ascending; horizontally) indicate significant differences for the respective metabolite levels between each defined reproductive period; Independent-Samples Kruskall Wallis test with post hoc Dunn’s comparison; significant if p < 0.05.
(DOCX)

S2 Table Descriptives for Tian Tian’s 2014 reproductive cycle (SB569): USpG-, creatinine-corrected and raw metabolite concentration, USpG-values and creatinine concentrations in urine, faecal output and bodyweight. Stdev = standard deviation; n = number of samples; USpG = urinary specific gravity; cr = creatinine. Different superscripts (a-d; ascending; horizontally) indicate significant differences for the respective metabolite levels between each defined reproductive period; Independent-Samples Kruskall Wallis test with post hoc Dunn’s comparison; significant if p < 0.05.
(DOCX)

S3 Table Descriptives for Tian Tian’s 2015 reproductive cycle (SB569): USpG-, creatinine-corrected and raw metabolite concentration, USpG-values and creatinine concentrations in urine, faecal output and bodyweight. Stdev = standard deviation; n = number of samples; USpG = urinary specific gravity; cr = creatinine. Different superscripts (a-d; ascending; horizontally) indicate significant differences for the respective metabolite levels between each defined reproductive period; Independent-Samples Kruskall Wallis test with post hoc Dunn’s comparison; significant if p < 0.05.
(DOCX)

S4 Table Descriptives for Tian Tian’s 2016 reproductive cycle (SB569): USpG-, creatinine-corrected and raw metabolite concentration, USpG-values and creatinine concentrations in urine, faecal output and bodyweight. Stdev = standard deviation; n = number of samples; USpG = urinary specific gravity; cr = creatinine. Different superscripts (a-d; ascending; horizontally) indicate significant differences for the respective metabolite levels between each defined reproductive period; Independent-Samples Kruskall Wallis test with post hoc Dunn’s comparison; significant if p < 0.05.
(DOCX)

S5 Table Descriptives for Tian Tian’s 2017 reproductive cycle (SB569): USpG-, creatinine-corrected and raw metabolite concentration, USpG-values and creatinine concentrations in urine, faecal output and bodyweight. Stdev = standard deviation; n = number of samples; USpG = urinary specific gravity; cr = creatinine. Different superscripts (a-d; ascending; horizontally) indicate significant differences for the respective metabolite levels between each defined reproductive period; Independent-Samples Kruskall Wallis test with post hoc Dunn’s comparison; significant if p < 0.05.
(DOCX)
