# Supplementary figures and images for: Urinary specific gravity as an alternative for the normalisation of endocrine metabolite concentrations in giant panda (Ailuropoda melanoleuca) reproductive monitoring
Source: PLoS One. 2018 Jul 26;13(7):e0201420. doi: 10.1371/journal.pone.0201420 (PMC6062134; doi:10.1371/journal.pone.0201420)

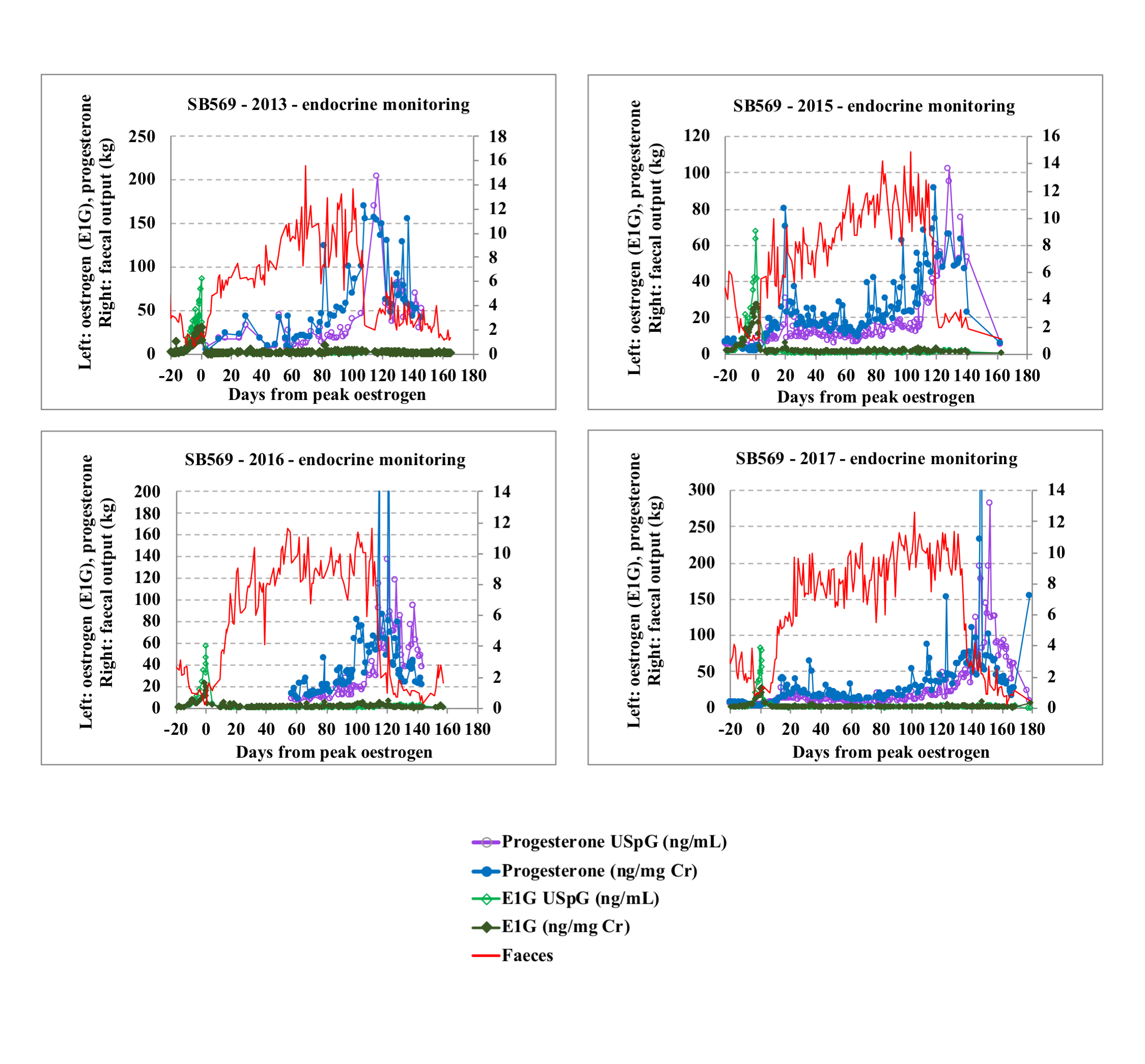

Supplement: S1 Fig — (TIF) [file pone.0201420.s001.tif]

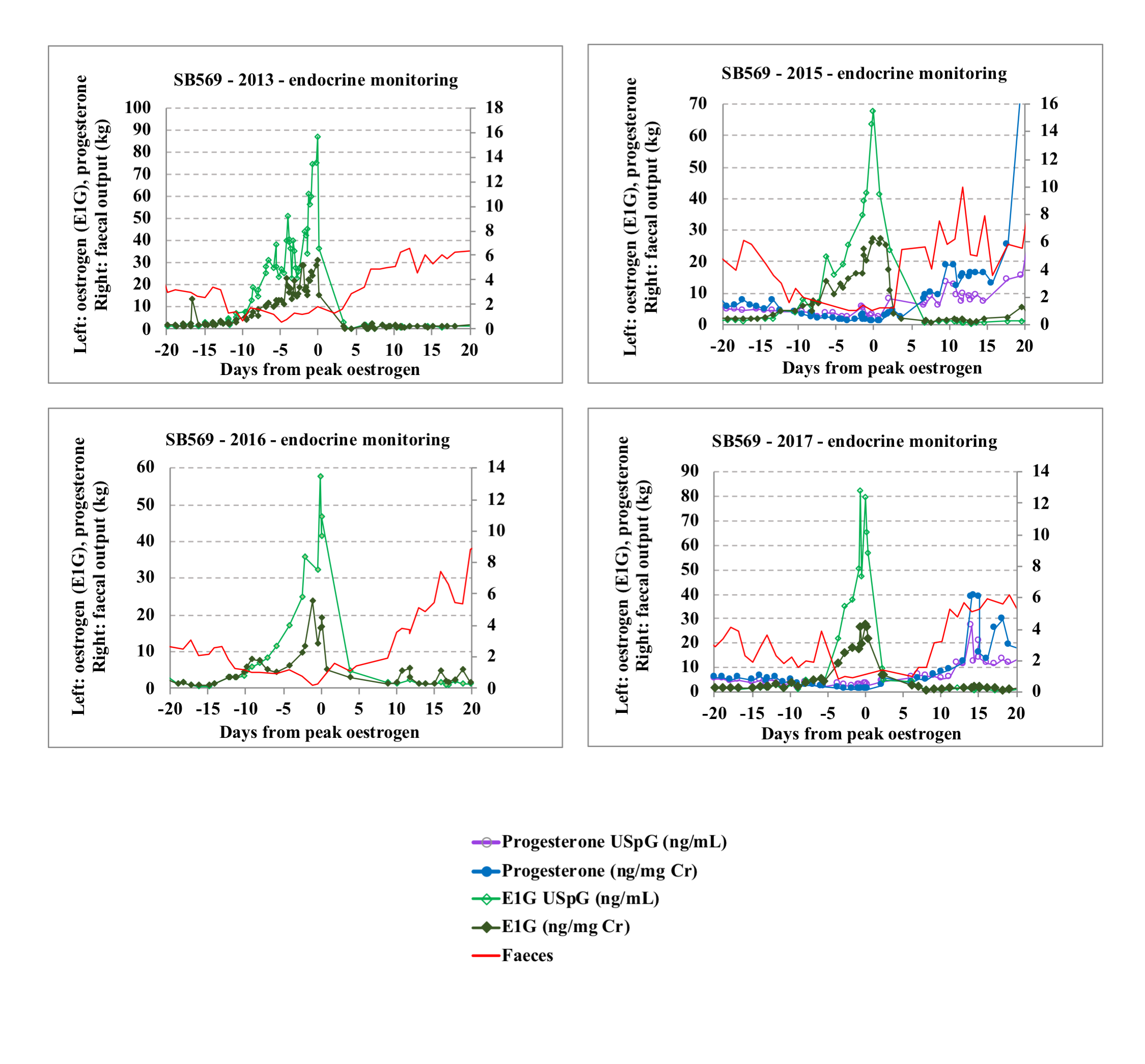

Supplement: S2 Fig — (TIF) [file pone.0201420.s002.tif]
