# Supplementary material for: Urinary specific gravity as an alternative for the normalisation of endocrine metabolite concentrations in giant panda (Ailuropoda melanoleuca) reproductive monitoring
Source: PLoS One. 2018 Jul 26;13(7):e0201420. doi: 10.1371/journal.pone.0201420 (PMC6062134; doi:10.1371/journal.pone.0201420)
Supplement: S1 Table — Stdev = standard deviation; n = number of samples; USpG = urinary specific gravity; cr = creatinine. Different superscripts (a-d; ascending; horizontally) indicate significant differences for the respective metabolite levels between each defined reproductive period; Independent-Samples Kruskall Wallis test with post hoc Dunn’s comparison; significant if p < 0.05. (DOCX) [file pone.0201420.s003.docx]

**S1 Table. Descriptives for Tian Tian’s 2013 reproductive cycle (SB569): USpG-, creatinine-corrected and raw metabolite concentration, USpG-values and creatinine concentrations in urine, faecal output and bodyweight.**

|  | **Anoestrus** | | **Pro-oestrus** | | **Postoestrus** | | **Primary P4 rise** | | **Secondary P4 rise** | |
| --- | --- | --- | --- | --- | --- | --- | --- | --- | --- | --- |
|  | D-63-D-14 | | D-13-D0 | | D0/1-D7 | | D8-D98 | | D99-D165 | |
|  | **Mean (stdev)** | **Median**  **(range)** | **Mean (stdev)** | **Median (range)** | **Mean (stdev)** | **Median**  **(range)** | **Mean (stdev)** | **Median (range)** | **Mean (stdev)** | **Median**  **(range)** |
| **Oestrogens** | **(n=60/78)** | | **(n=46/57)** | | **(n=12/20)** | | **(n=74/85)** | | **(n=51/65)** | |
| USpG  (ng/mL) | 1.51  (0.46) | 1.48  (0.74-3.10)^a^ | 29.65 (20.85) | 27.60  (2.23-86.93)^b^ | 4.30  (10.12) | 1.72  (0.31-36.31)^a^ | 1.27  (0.40) | 1.17  (0.48-2.94)^a^ | 1.63  (0.64) | 1.53  (0.76-4.20)^a^ |
| Creatinine (ng/mg Cr) | 1.97  (0.52) | 1.96  (0.93-3.37)^a^ | 13.23 (7.70) | 13.01  (2.33-31.20)^b^ | 2.21  (4.29) | 1.16  (0.28-15.67)^a^ | 1.71  (0.84) | 1.71  (0.78-5.15)^a^ | 1.72  (0.78) | 1.51  (0.63-4.19)^a^ |
| Raw  (ng/ mL) | 3.16  (2.56) | 2.64  (0.44-13.19)^a^ | 92.07 (82.98) | 78.81  (6.18-315.1)^b^ | 14.43 (35.57) | 5.41  (1.07-127.1)^a^ | 1.36  (0.58) | 1.28  (0.36-3.27)^a^ | 2.78  (2.07) | 2.12  (0.61-12.59)^a^ |
| **Progesterone** |  | |  | |  | | **(n=31/85)** | | **(n=22/65)** | |
| USpG  (ng/mL) |  |  |  |  |  |  | 18.19 (8.57) | 17.52  (5.14-43.68)^a^ | 67.80  (41.35) | 54.72  (29.43-203.1)^b^ |
| Creatinine (ng/mg Cr) |  |  |  |  |  |  | 34.21 (25.63) | 25.01  (5.59-124.1)^a^ | 89.13  (41.73) | 72.95  (40.80-169.1)^b^ |
| Raw  (ng/ mL) |  |  |  |  |  |  | 16.50 (6.79) | 15.77  (5.17-35.80)^a^ | 106.04 (62.48) | 99.37  (34.85-289.5)^b^ |
| **Ceruloplasmin** |  | |  | |  | | **(n=31/85)** | | **(n=30/65)** | |
| USpG  (ng/mL) |  |  |  |  |  |  | 34.30 (19.33) | 30.36  (2.07-82.40)^b^ | 13.00  (9.09) | 11.45  (0.53-37.89)^a^ |
| Creatinine (ng/mg Cr) |  |  |  |  |  |  | 62.82 (45.79) | 52.39  (7.26-248.4)^b^ | 13.48  (12.70) | 10.31  (0.00-62.63)^a^ |
| Raw  (ng/ mL) |  |  |  |  |  |  | 32.02 (16.86) | 29.10  (2.59-91.80)^b^ | 17.01  (9.57) | 16.70  (0.00-45.78)^a^ |
| **PGFM** |  | |  | |  | | **(n=16/85)** | | **(n=38/65)** | |
| USpG  (ng/mL) |  |  |  |  |  |  | 3.06  (1.90) | 2.44  (1.12-7.92)^a^ | 14.77  (15.94) | 9.59  (1.89-92.66)^b^ |
| Creatinine (ng/mg Cr) |  |  |  |  |  |  | 4.78  (2.04) | 4.38  (1.91-8.39)^a^ | 13.46  (11.94) | 10.10  (2.31-69.67)^b^ |
| Raw  (ng/ mL) |  |  |  |  |  |  | 2.92  (1.48) | 2.50  (0.97-6.39)^a^ | 25.31  (30.80) | 16.59  (1.65-171.5)^b^ |
| **USpG** | **(n= 60/78)** | | **(n= 46/57 )** | | **(n= 12/20)** | | **(n= 78/85)** | | **(n= 53/65)** | |
| USpG | 1.016 (0.009) | 1.016  (1.003-1.034)^b^ | 1.023 (0.006) | 1.023  (1.005-1.037)^c^ | 1.026 (0.004) | 1.028  (1.020-1.030)^c^ | 1.009 (0.004) | 1.008  (1.000-1.018)^a^ | 1.013  (0.006) | 1.013  (1.001-1.030)^a,b^ |
| **Cr** | **(n= 77/78)** | | **(n= 57/57)** | | **(n= 18/20)** | | **(n= 85/85)** | | **(n= 65/65)** | |
| Creatinine (mg/mL) | 1.66  (1.13) | 1.67  (0.22-4.64)^a^ | 6.11  (2.80) | 2.80  (1.10-12.47)^c^ | 4.76  (1.42) | 4.71  (2.78-8.11)^b^ | 0.77  (0.47) | 0.66  (0.01-2.16)^a^ | 1.69  (1.10) | 1.63  (0.00-5.11)^a^ |
| **Faeces** | **(n= 48/78)** | | **(n= 14/57)** | | **(n= 5/20)** | | **(n= 70/85 )** | | **(n= 56/65 )** | |
| Faeces  (kg) | 4.42  (1.11) | 4.35  (2.60-6.60)^b^ | 1.39  (0.63) | 1.30  (0.60-3.20)^a^ | 2.82  (1.45) | 2.90  (1.30-4.90)^a,b^ | 8.30  (2.43) | 7.55  (4.60-15.60)^c^ | 3.69  (2.82) | 1.55  (1.10-13.70)^a,b^ |
| **Bodyweight** | **(n= 48/78)** | | **(n= 6/57)** | | **(n= 5/20)** | | **(n= 70/85)** | | **(n= 23/65)** | |
| Bodyweight (kg) | 105.7  (0.9) | 105.8  (103.7-108.5)^b,c^ | 103.2 (2.02) | 104.1  (99.7-105.3)^a,b^ | 100.00 (1.8) | 98.8  (98.5-102.3)^a^ | 109.7 (5.18) | 111.2  (100.6-118.4)^c^ | 110.7  (4.0) | 109.1  (105.0-117.9)^c^ |

Stdev = standard deviation; n = number of samples; USpG = urinary specific gravity; cr = creatinine. Different superscripts (a-d; ascending; horizontally) indicate significant differences for the respective metabolite levels between each defined reproductive period; Independent-Samples Kruskall Wallis test with post hoc Dunn’s comparison; significant if p < 0.05.
