# Supplementary material for: Urinary specific gravity as an alternative for the normalisation of endocrine metabolite concentrations in giant panda (Ailuropoda melanoleuca) reproductive monitoring
Source: PLoS One. 2018 Jul 26;13(7):e0201420. doi: 10.1371/journal.pone.0201420 (PMC6062134; doi:10.1371/journal.pone.0201420)
Supplement: S2 Table — Stdev = standard deviation; n = number of samples; USpG = urinary specific gravity; cr = creatinine. Different superscripts (a-d; ascending; horizontally) indicate significant differences for the respective metabolite levels between each defined reproductive period; Independent-Samples Kruskall Wallis test with post hoc Dunn’s comparison; significant if p < 0.05. (DOCX) [file pone.0201420.s004.docx]

**S2 Table. Descriptives for Tian Tian’s 2014 reproductive cycle (SB569): USpG-, creatinine-corrected and raw metabolite concentration, USpG-values and creatinine concentrations in urine, faecal output and bodyweight.**

|  | **Anoestrus** | | **Pro-oestrus** | | **Postoestrus** | | **Primary P4 rise** | | **Secondary P4 rise** | |
| --- | --- | --- | --- | --- | --- | --- | --- | --- | --- | --- |
|  | D-107-D-14 | | D-13-D0 | | D0/1-D7 | | D8-D97 | | D98-D166 | |
|  | **Mean (stdev)** | **Median**  **(range)** | **Mean (stdev)** | **Median**  **(range)** | **Mean (stdev)** | **Median (range)** | **Mean (stdev)** | **Median**  **(range)** | **Mean (stdev)** | **Median**  **(range)** |
| **Oestrogens** | **(n=64/87)** | | **(n=9/22)** | | **(n=3/11)** | | **(n=77/86)** | | **(n=56/69)** | |
| USpG  (ng/mL) | 1.61  (0.52) | 1.50  (0.63-3.34)^a^ | 42.02 (11.55) | 42.40  (25.00-63.54)^c^ | 10.84 (10.66) | 8.45  (1.58-22.50)^b^ | 1.06  (0.37) | 0.96  (0.51-2.27)^a^ | 1.17  (0.57) | 1.04  (0.22-2.83)^a^ |
| Creatinine  (ng/mg Cr) | 2.77  (0.73) | 2.61  (1.54-5.85)^a^ | 22.33  (4.08) | 21.80  (15.84-28.32)^c^ | 9.10  (6.81) | 11.67  (1.39-14.25)^b^ | 1.65  (0.48) | 1.70  (0.62-3.31)^a^ | 1.20  (0.56) | 1.07  (0.25-3.34)^a^ |
| Raw  (ng/ mL) | 1.29  (1.07) | 1.01  (0.40-5.85)^a^ | 102.96 (71.15) | 84.80  (31.72-222.4)^b^ | 40.13 (46.58) | 23.24  (4.34-92.80)^b^ | 0.98  (0.56) | 0.80  (0.26-2.60)^a^ | 1.65  (1.18) | 1.24  (0.17-5.49)^a^ |
| **Progesterone** | **(n=21/87)** | | **(n=9/22)** | | **(n=3/11)** | | **(n=77/85)** | | **(n=56/69)** | |
| USpG  (ng/mL) | 5.44  (1.34) | 5.21  (3.10-7.59)^a^ | 2.42  (0.94) | 2.05  (1.81-4.83)^a^ | 3.63  (1.47) | 4.29  (1.95-4.66)^a^ | 15.43 (4.56) | 15.00  (6.32-28.00)^a^ | 47.66 (32.41) | 39.16  (3.26-143.5)^b^ |
| Creatinine  (ng/mg Cr) | 8.48  (1.84) | 8.90  (5.33-13.03)^a,b^ | 1.28  (0.30) | 1.22  (0.99-1.97)^a^ | 3.17  (0.80) | 2.72  (2.69-4.09)^a^ | 24.84 (9.93) | 21.44  (13.44-53.73)^b^ | 50.67 (32.28) | 43.21  (1.82-138.8)^c^ |
| Raw  (ng/ mL) | 4.73  (2.27) | 4.14  (1.95-9.63)^a^ | 6.09  (4.91) | 4.10  (1.55-16.89)^a^ | 11.95 (6.20) | 12.81  (5.36-17.68)^a^ | 13.73 (6.14) | 12.95  (5.22-35.25)^a^ | 62.19 (47.53) | 43.40  (5.29-179.4)^b^ |
| **Ceruloplasmin** |  | |  | |  | | **(n=77/85)** | | **(n=56/69)** | |
| USpG  (ng/mL) |  |  |  |  |  |  | 40.75 (17.74) | 39.00  (10.78-92.73)^b^ | 12.23 (14.12) | 8.19  (0.00-74.00)^a^ |
| Creatinine  (ng/mg Cr) |  |  |  |  |  |  | 67.76 (36.55) | 63.30  (17.28-172.2)^b^ | 15.59 (26.50) | 7.76  (0.00-174.5)^a,b^ |
| Raw  (ng/ mL) |  |  |  |  |  |  | 33.89 (14.51) | 33.03  (6.74-104.3)^b^ | 14.17 (13.18) | 9.23  (0.00-56.57)^a^ |
| **PGFM** |  | |  | |  | | **(n=19/85)** | | **(n=56/69)** | |
| USpG  (ng/mL) |  |  |  |  |  |  | 4.49  (2.01) | 3.92  (1.85-10.09)^a^ | 15.95 (15.68) | 11.71  (2.21-93.95)^b^ |
| Creatinine  (ng/mg Cr) |  |  |  |  |  |  | 9.51  (6.74) | 6.91  (3.90-24.20)^a^ | 15.45 (11.11) | 11.69  (3.28-54.37)^b^ |
| Raw  (ng/ mL) |  |  |  |  |  |  | 3.36  (1.94) | 3.05  (0.92-7.60)^a^ | 24.72 (29.55) | 14.29  (2.21-148.9)^b^ |
| **USpG** | **(n= 79/87)** | | **(n= 10/22)** | | **(n= 4/11)** | | **(n= 81/85)** | | **(n= 58/69)** | |
| USpG | 1.005 (0.005) | 1.004  (1.000-1.026)^a^ | 1.017 (0.010) | 1.016  (1.002-1.033)^b^ | 1.020 (0.013) | 1.022  (1.001-1.033)^b^ | 1.007 (0.003) | 1.006  (1.002-1.017)^a^ | 1.011 (0.005) | 1.011  (1.002-1.021)^a^ |
| **Cr** | **(n= 87/87)** | | **(n= 22/22)** | | **(n= 11/11)** | | **(n= 85/85)** | | **(n= 69/69)** | |
| Creatinine (mg/mL) | 0.42  (0.41) | 0.31  (0.00-2.45)^a^ | 3.84  (3.12) | 3.36  (0.02-9.51)^b^ | 4.61  (3.50) | 4.89  (0.05-10.13)^b^ | 0.61  (0.41) | 0.51  (0.07-2.09)^a^ | 1.24  (0.86) | 1.01  (0.00-4.31)^a^ |
| **Faeces** | **(n= 86/87 )** | | **(n= 2/22)** | | **(n= 2/11)** | | **(n= 78/85)** | | **(n= 39/69)** | |
| Faeces  (kg) | 5.12  (0.97) | 5.20  (2.40-7.30)^a,b^ | 1.90  (0.71) | 1.90  (1.40-2.40)^a^ | 3.85  (0.21) | 3.85  (3.70-4.00)^a^ | 9.03  (2.05) | 8.9  (5.50-15.90)^b^ | 4.92  (3.52) | 3.20  (1.10-13.80)^a,b^ |
| **Bodyweight** | **(n= 86/87)** | | **(n= 2/22)** | | **(n= 2/11)** | | **(n= 78/85)** | | **(n= 22/69)** | |
| Bodyweight (kg) | 107.7  (1.2) | 107.6  (105.1-110.5)^a,b^ | 102.3  (3.3) | 102.3  (100.0-104.6)^a^ | 103.0  (1.4) | 103.0  (102.0-104.0)^a^ | 113.3 `(4.2) | 112.3  (105.1-119.2)^b,c^ | 116.2  (3.3) | 116.2  (106.4-120.7)^c^ |

Stdev = standard deviation; n = number of samples; USpG = urinary specific gravity; cr = creatinine. Different superscripts (a-d; ascending; horizontally) indicate significant differences for the respective metabolite levels between each defined reproductive period; Independent-Samples Kruskall Wallis test with post hoc Dunn’s comparison; significant if p < 0.05.
