# Supplementary material for: Urinary specific gravity as an alternative for the normalisation of endocrine metabolite concentrations in giant panda (Ailuropoda melanoleuca) reproductive monitoring
Source: PLoS One. 2018 Jul 26;13(7):e0201420. doi: 10.1371/journal.pone.0201420 (PMC6062134; doi:10.1371/journal.pone.0201420)
Supplement: S3 Table — Stdev = standard deviation; n = number of samples; USpG = urinary specific gravity; cr = creatinine. Different superscripts (a-d; ascending; horizontally) indicate significant differences for the respective metabolite levels between each defined reproductive period; Independent-Samples Kruskall Wallis test with post hoc Dunn’s comparison; significant if p < 0.05. (DOCX) [file pone.0201420.s005.docx]

**S3 Table. Descriptives for Tian Tian’s 2015 reproductive cycle (SB569): USpG-, creatinine-corrected and raw metabolite concentration, USpG-values and creatinine concentrations in urine, faecal output and bodyweight.**

|  | **Anoestrus** | | **Pro-oestrus** | | **Postoestrus** | | **Primary P4 rise** | | **Secondary P4 rise** | |
| --- | --- | --- | --- | --- | --- | --- | --- | --- | --- | --- |
|  | D-111-D-13 | | D-12-D0 | | D0/1-D7 | | D8-D111 | | D112-D163 | |
|  | **Mean (stdev)** | **Median (range)** | **Mean (stdev)** | **Median**  **(range)** | **Mean (stdev)** | **Median**  **(range)** | **Mean (stdev)** | **Median**  **(range)** | **Mean (stdev)** | **Median**  **(range)** |
| **Oestrogens** | **(n=75/77)** | | **(n=15/21)** | | **(n=4/9)** | | **(n=100/108)** | | **(n=19/23)** | |
| USpG  (ng/mL) | 1.49  (0.45) | 1.42  (0.61-3.67)^a^ | 24.57 (21.09) | 19.56  (4.24-68.20)^c^ | 16.99 (19.75) | 12.59  (0.98-41.81)^b^ | 0.88  (0.23) | 0.89  (0.35-1.45)^a^ | 1.35  (0.52) | 1.22  (0.47-2.56)^a^ |
| Creatinine  (ng/mg Cr) | 2.10  (0.53) | 2.02  (1.21-3.78)^a^ | 13.26 (7.89) | 11.81  (4.56-27.42)^b^ | 9.95 (11.48) | 6.35  (1.31-25.81)^b^ | 1.59  (0.66) | 1.49  (0.78-5.88)^a^ | 1.54  (0.62) | 1.42  (0.50-3.19)^a^ |
| Raw  (ng/ mL) | 1.47  (1.09) | 1.07  (0.31-5.65)^a^ | 56.99 (56.86) | 48.91  (8.06-204.0)^b^ | 50.80 (59.64) | 41.06  (0.86-120.2)^b^ | 0.79  (0.41) | 0.70  (0.20-2.36)^a^ | 1.58  (0.90) | 1.60  (0.30-3.47)^a^ |
| **Progesterone** | **(n=75/77)** | | **(n=15/21)** | | **(n=4/9)** | | **(n=100/108)** | | **(n=19/23)** | |
| USpG  (ng/mL) | 6.35  (2.01) | 5.88  (2.77-14.08)^a^ | 3.68  (1.01) | 3.62  (2.39-5.80)^a^ | 5.93  (2.57) | 6.56  (2.29-8.29)^a^ | 11.93 (4.03) | 11.14  (5.66-30.50)^a^ | 49.10 (22.78) | 48.15  (4.77-101.8)^b^ |
| Creatinine  (ng/mg Cr) | 9.42  (4.09) | 7.99  (4.46-24.38)^a^ | 2.61  (1.11) | 2.33  (1.25-4.66)^a^ | 5.79  (3.83) | 6.05  (1.42-9.63)^a^ | 22.26 (12.35) | 18.90  (8.98-79.72)^b^ | 53.67 (18.59) | 52.70  (5.17-91.08)^c^ |
| Raw  (ng/ mL) | 6.01  (4.14) | 6.01  (1.04-24.60)^a^ | 8.11  (3.49) | 8.11  (3.03-15.22)^a^ | 11.73 (10.86) | 6.75  (5.44-27.99)^a^ | 10.37 (5.47) | 9.01  (3.40-38.14)^a^ | 57.00 (35.56) | 62.46  (10.14-141.9)^b^ |
| **Ceruloplasmin** | **(n=32/77)** | | **(n=15/21)** | | **(n=4/9)** | | **(n=96/108)** | | **(n=19/23)** | |
| USpG  (ng/mL) | 19.74 (17.53) | 15.87  (0.13-90.60)^a^ | 10.44 (9.33) | 9.92  (0.08-28.87)^a^ | 23.96 (9.71) | 27.74  (9.66-30.70)^a^ | 36.51 (31.08) | 27.39  (0.32-156.1)^a^ | 21.58 (25.21) | 12.73  (0.33-82.00)^a^ |
| Creatinine  (ng/mg Cr) | 25.64 (22.57) | 21.00  (0.13-111.6)^a,b^ | 6.55  (5.77) | 6.15  (0.05-15.54)^a^ | 25.63 (18.39) | 27.61  (4.44-42.88)^a,b^ | 70.95 (72.95) | 41.85  (0.48-371.2)^b^ | 33.70 (47.76) | 15.95  (0.23-157.8)^a,b^ |
| Raw  (ng/ mL) | 20.08 (16.04) | 16.60  (0.10-66.20)^a^ | 21.57 (19.22) | 25.50  (0.10-61.10)^b^ | 41.08 (23.07) | 31.65  (25.60-75.40)^a^ | 27.78 (20.26) | 25.27  (0.20-136.6)^a^ | 17.61 (15.89) | 14.35  (0.50-57.02)^a^ |
| **PGFM** |  | | **(n=12/21)** | | **(n=4/9)** | | **(n=55/108)** | | **(n=18/23)** | |
| USpG  (ng/mL) |  |  | 26.22 (14.43) | 21.84  (7.79-53.87)^c^ | 10.40 (7.28) | 9.24  (3.96-19.13)^a,b^ | 5.12  (2.79) | 4.01  (2.11-15.30)^a^ | 19.99 (22.01) | 13.95  (3.42-80.21)^b,c^ |
| Creatinine  (ng/mg Cr) |  |  | 14.70 (5.07) | 14.21  (7.82-22.16)^a,b^ | 7.52  (2.89) | 6.37  (5.53-11.81)^a^ | 9.66  (6.52) | 7.42  (2.99-26.41)^a,b^ | 18.95 (15.88) | 14.25  (6.01-53.22)^b^ |
| Raw  (ng/ mL) |  |  | 63.05 (47.01) | 54.94  (13.63-160.6)^b^ | 27.30 (27.04) | 25.12  (3.96-55.01)^a^ | 4.98  (4.67) | 3.12  (1.13-21.03)^a^ | 23.51 (27.21) | 17.52  (1.28-115,0)^a^ |
| **USpG** | **(n= 76/77)** | | **(n= 16/21)** | | **(n= 5/9)** | | **(n= 105/108)** | | **(n= 23/23)** | |
| USpG | 1.008 (0.006) | 1.006  (1.001-1.028)^a,b^ | 1.017 (0.007) | 1.016  (1.002-1.026)^c^ | 1.013 (0.109) | 1.008  (1.002-1.027)^b,c^ | 1.007 (0.003) | 1.006  (1.001-1.017)^a^ | 1.008 (0.005) | 1.008  (1.001-1.017)^a,b^ |
| **Cr** | **(n= 77/77)** | | **(n= 21/21)** | | **(n= 9/9)** | | **(n= 101/108)** | | **(n= 20/23)** | |
| Creatinine (mg/mL) | 0.76  (0.61) | 0.51  (0.08-2.66)^a^ | 3.67  (2.32) | 3.07  (0.00-9.51)^b^ | 3.67  (2.79) | 3.66  (0.20-7.34)^b^ | 0.53 `(0.28) | 0.48  (0.09-1.52)^a^ | 1.17  (0.86) | 1.15  (0.18-3.54)^a^ |
| **Faeces** | **(n= 76/77)** | | **(n= 8/21)** | | **(n= 4/9)** | | **(n= 95/108)** | | **(n= 22/23)** | |
| Faeces  (kg) | 4.37  (1.14) | 4.45  (1.90-7.50)^a,b^ | 1.70  (0.77) | 1.50  (1.00-3.00)^a^ | 3.41  (2.53) | 3.37  (1.20-5.70)^a,b^ | 9.17  (2.32) | 9.30  (3.60-14.90)^b^ | 4.94 `(3.74) | 3.05  (1.10-12.80)^c^ |
| **Bodyweight** | **(n= 76/77)** | | **(n= 6/21)** | | **(n= 1/9)** | | **(n= 96/108)** | | **(n= 16/23)** | |
| Bodyweight  (kg) | 104.6  (0.9) | 104.7  (102.0-106.5)^a^ | 104.9  (1.7) | 104.0  (103.6-107.0)^a^ |  |  | 109.8  (5.6) | 108.5  (101.2-120.4)^b^ | 116.6  (5.3) | 116.6  (112.0-119.9)^c^ |

Stdev = standard deviation; n = number of samples; USpG = urinary specific gravity; cr = creatinine. Different superscripts (a-d; ascending; horizontally) indicate significant differences for the respective metabolite levels between each defined reproductive period; Independent-Samples Kruskall Wallis test with post hoc Dunn’s comparison; significant if p < 0.05.
