# Supplementary material for: Urinary specific gravity as an alternative for the normalisation of endocrine metabolite concentrations in giant panda (Ailuropoda melanoleuca) reproductive monitoring
Source: PLoS One. 2018 Jul 26;13(7):e0201420. doi: 10.1371/journal.pone.0201420 (PMC6062134; doi:10.1371/journal.pone.0201420)
Supplement: S4 Table — Stdev = standard deviation; n = number of samples; USpG = urinary specific gravity; cr = creatinine. Different superscripts (a-d; ascending; horizontally) indicate significant differences for the respective metabolite levels between each defined reproductive period; Independent-Samples Kruskall Wallis test with post hoc Dunn’s comparison; significant if p < 0.05. (DOCX) [file pone.0201420.s006.docx]

**S4 Table. Descriptives for Tian Tian’s 2016 reproductive cycle (SB569): USpG-, creatinine-corrected and raw metabolite concentration, USpG-values and creatinine concentrations in urine, faecal output and bodyweight.**

|  | **Anoestrus** | | **Pro-oestrus** | | **Postoestrus** | | **Primary P4 rise** | | **Secondary P4 rise** | |
| --- | --- | --- | --- | --- | --- | --- | --- | --- | --- | --- |
|  | D-208-D-10 | | D-9-D0 | | D0/1-D7 | | D8-D109 | | D110-D157 | |
|  | **Mean (stdev)** | **Median**  **(range)** | **Mean (stdev)** | **Median**  **(range)** | **Mean (stdev)** | **Median (range)** | **Mean (stdev)** | **Median**  **(range)** | **Mean (stdev)** | **Median (range)** |
| **Oestrogens** | **(n=167/192)** | | **(n=11/12)** | |  | | **(n=99/122)** | | **(n=37/44)** | |
| USpG  (ng/mL) | 1.47  (0.59) | 1.45  (0.55-4.58)^a^ | 26.18 (17.85) | 24.93  (5.62-57.52)^b^ |  |  | 1.09  (0.36) | 1.08  (0.43-2.14)^a^ | 2.09  (0.66) | 2.16  (0.61-3.49)^a^ |
| Creatinine (ng/mg Cr) | 1.90  (1.19) | 1.62  (0.66-8.80)^a^ | 10.63 (5.07) | 9.59  (4.35-19.29)^b^ |  |  | 1.90  (1.03) | 1.62  (0.52-5.95)^a^ | 1.94  (1.41) | 1.41  (0.27-7.23)^a^ |
| Raw  (ng/ mL) | 1.72  (1.13) | 1.39  (0.34-8.50)^a^ | 76.85 (53.69) | 88.35  (12.64-172.6)^b^ |  |  | 0.93  (0.66) | 0.78  (0.31-4.43)^a^ | 2.89  (1.68) | 3.08  (0.62-6.94)^a^ |
| **Progesterone** |  | |  | |  | | **(n=42/122)** | | **(n=32/44)** | |
| USpG  (ng/mL) |  |  |  |  |  |  | 14.17 (6.26) | 11.87  (6.83-31.74)^a^ | 67.44 (26.04) | 60.11  (29.31-136.6)^b^ |
| Creatinine (ng/mg Cr) |  |  |  |  |  |  | 27.38 (17.46) | 21.90  (8.03-80.67)^a^ | 63.63 (65.62) | 40.77  (21.55-283.0)^b^ |
| Raw  (ng/ mL) |  |  |  |  |  |  | 9.67  (4.64) | 9.23  (2.67-23.18)^a^ | 74.58 (29.47) | 82.24  (20.64-118.5)^b^ |
| **Ceruloplasmin** | **(n=125/192)** | | **(n=11/12)** | |  | | **(n=100/122)** | | **(n=38/44)** | |
| USpG  (ng/mL) | 7.26 (11.10) | 2.60  (0.05-57.80)^a^ | 11.39 (9.38) | 11.82  (0.13-30.31)^a^ |  |  | 37.07 (28.61) | 30.56  (0.18-138.3)^b^ | 7.37  (7.37) | 4.68  (0.07-28.32)^a^ |
| Creatinine (ng/mg Cr) | 12.82 (26.39) | 3.45  (0.07-189.4)^a^ | 5.19 (3.79) | 4.84  (0.04-12.22)^a^ |  |  | 66.46 (58.44) | 51.16  (0.31-310.9)^a^ | 7.92 (11.25) | 3.62  (0.09-57.47)^a^ |
| Raw  (ng/ mL) | 7.68 (10.11) | 3.90  (0.10-52.00)^a^ | 31.23 (24.58) | 29.55  (0.50-72.90)^b^ |  |  | 26.35 (19.18) | 23.50  (0.20-103.70)^a,b^ | 8.69  (9.47) | 5.82  (0.10-47.70)^a^ |
| **PGFM** | **(n=4/192)** | | **(n=11/12)** | |  | | **(n=28/122)** | | **(n=37/44)** | |
| USpG  (ng/mL) | 12.71 (6.74) | 10.74  (7.07-22.30)^a^ | 36.72 (22.84) | 22.67  (14.41-83.79)^a,b^ |  |  | 7.63  (4.42) | 6.52  (2.50-16.41)^a^ | 77.30 (65.73) | 58.49  (2.42-308.1)^b^ |
| Creatinine (ng/mg Cr) | 14.12 (6.44) | 12.76  (8.89-22.09)^a^ | 16.92 (8.82) | 17.23  (7.95-32.13)^a^ |  |  | 13.06 (9.30) | 9.79  (4.36-40.49)^a^ | 57.31 (54.83) | 34.75  (4.82-263.5)^a^ |
| Raw  (ng/ mL) | 26.85 (24.40) | 19.94  (6.19-61.33)^a^ | 113.24 (84.11) | 68.00  (36.03-261.8)^a^ |  |  | 6.97  (7.59) | 5.37  (1.25-39.00)^a^ | 108.07 (85.04) | 90.69  (1.51-299.9)^a^ |
| **USpG** | **(n= 190/192)** | | **(n= 11/12)** | | **(n= 4/4)** | | **(n= 120/122)** | | **(n= 41/44)** | |
| USpG | 1.009 (0.005) | 1.008  (1.001-1.027)^b^ | 1.023 (0.005) | 1.024  (1.017-1.032)^c^ | 1.002 (0.002) | 1.002  (1.000-1.004)^a^ | 1.006 (0.004) | 1.005  (1.001-1.024)^a,b^ | 1.011 (0.007) | 1.010  (1.001-1.028)^b^ |
| **Cr** | **(n= 192/192)** | | **(n= 12/12)** | | **(n= 4/4)** | | **(n= 122/122)** | | **(n= 44/44)** | |
| Creatinine (mg/mL) | 0.92  (0.64) | 0.80  (0.00-3.10)^a,b^ | 7.01 (10.71) | 8.01  (1.58-11.64)^c^ | 0.29 (0.38) | 0.14  (0.03-0.84)^a^ | 0.50  (0.48) | 0.39  (0.00-3.50)^a^ | 2.07  (1.73) | 2.00  (0.01-8.48)^b^ |
| **Faeces** | **(n= 161/192)** | | **(n=7/12)** | | **(n= 3/4)** | | **(n= 92/122)** | | **(n= 36/44)** | |
| Faeces  (kg) | 2.60  (1.06) | 2.30  (0.90-5.20)^a^ | 0.77  (0.38) | 0.90  (0.20-1.20)^a^ | 1.37 (0.25) | 1.40  (1.10-1.60)^a^ | 8.28  (1.98) | 8.60  (1.90-11.60)^b^ | 2.42  (2.60) | 1.25  (0.30-11.60)^a^ |
| **Bodyweight** | **(n= 147/192)** | |  | |  | | **(n= 90/122)** | | **(n= 9/44)** | |
| Bodyweight (kg) | 109.6  (0.9) | 109.5  (107.8-112.0)^a^ |  |  |  |  | 112.9  (3.8) | 113.2  (104.0-119.2)^b^ | 115.7  (3.3) | 116.8  (107.5-119.0)^c^ |

Stdev = standard deviation; n = number of samples; USpG = urinary specific gravity; cr = creatinine. Different superscripts (a-d; ascending; horizontally) indicate significant differences for the respective metabolite levels between each defined reproductive period; Independent-Samples Kruskall Wallis test with post hoc Dunn’s comparison; significant if p < 0.05.
