# Supplementary material for: Urinary specific gravity as an alternative for the normalisation of endocrine metabolite concentrations in giant panda (Ailuropoda melanoleuca) reproductive monitoring
Source: PLoS One. 2018 Jul 26;13(7):e0201420. doi: 10.1371/journal.pone.0201420 (PMC6062134; doi:10.1371/journal.pone.0201420)
Supplement: S5 Table — Stdev = standard deviation; n = number of samples; USpG = urinary specific gravity; cr = creatinine. Different superscripts (a-d; ascending; horizontally) indicate significant differences for the respective metabolite levels between each defined reproductive period; Independent-Samples Kruskall Wallis test with post hoc Dunn’s comparison; significant if p < 0.05. (DOCX) [file pone.0201420.s007.docx]

**S5 Table. Descriptives for Tian Tian’s 2017 reproductive cycle (SB569): USpG-, creatinine-corrected and raw metabolite concentration, USpG-values and creatinine concentrations in urine, faecal output and bodyweight.**

|  | **Anoestrus** | | **Pro-oestrus** | | **Postoestrus** | | **Primary P4 rise** | | **Secondary P4 rise** | |
| --- | --- | --- | --- | --- | --- | --- | --- | --- | --- | --- |
|  | D-104-D-9 | | D-8-D0 | | D1-D7 | | D9-D128 | | D129-D179 | |
|  | **Mean (stdev)** | **Median**  **(range)** | **Mean (stdev)** | **Median (range)** | **Mean (stdev)** | **Median**  **(range)** | **Mean (stdev)** | **Median**  **(range)** | **Mean (stdev)** | **Median**  **(range)** |
| **Oestrogens** | **(n= 83/92)** | | **(n= 11/11)** | | **(n= 6/6)** | | **(n= 97/125)** | | **(n= 40/46)** | |
| USpG (ng/mL) | 1.17  (0.48) | 1.07  (0.56-3.36)^a^ | 33.94 (28.95) | 34.96  (4.04-82.39)^c^ | 23.80 (28.92) | 7.27  (2.35-65.12)^b^ | 0.99  (0.42) | 0.90  (0.36-3.21)^a^ | 1.74  (0.69) | 1.66  (0.28-3.20)^a^ |
| Creatinine (ng/mg Cr) | 1.91  (0.67) | 1.75  (1.02-4.99)^a^ | 14.08 (8.72) | 16.16  (3.99-27.33)^c^ | 11.05 (10.44) | 6.74  (1.91-26.71)^b^ | 1.68  (0.69) | 1.59  (0.60-4.46)^a^ | 1.63  (1.43) | 1.36  (0.36-8.98)^a^ |
| Raw  (ng/ mL) | 1.24  (0.78) | 1.01  (0.34-4.55)^a^ | 107.00 (114.90) | 65.55  (11.12-350.1)^b^ | 79.26 (108.66) | 16.03  (1.85-244.3)^b^ | 0.76  (0.51) | 0.64  (0.17-3.60)^a^ | 3.34  (2.55) | 2.41  (0.42-9.60)^a^ |
| **Progesterone** | **(n= 80/92)** | | **(n= 11/11)** | | **(n= 6/6)** | | **(n= 97/125)** | | **(n= 40/46)** | |
| USpG (ng/mL) | 4.22  (1.15) | 4.26  (1.39-6.60)^a^ | 2.69  (0.48) | 2.67  (1.97-3.59)^a^ | 4.56  (1.51) | 4.28  (2.92-6.72)^a^ | 13.15 (6.76) | 11.5  (5.35-47.95)^a^ | 82.30 (53.48) | 69.93  (7.80-280.5)^b^ |
| Creatinine (ng/mg Cr) | 7.33  (3.23) | 6.27  (2.89-21.89)^a^ | 1.57  (0.66) | 1.29  (0.93-2.86)^a^ | 3.45  (2.09) | 3.37  (1.11-6.11)^a^ | 24.41 (19.18) | 18.31  (4.88-152.4)^a^ | 73.55 (90.69) | 57.79  (14.64-578.9)^b^ |
| Raw  (ng/ mL) | 4.38  (2.21) | 3.98  (0.57-13.45)^a^ | 7.86  (2.57) | 8.02  (5.22-12.31)^a^ | 11.00 (5.72) | 10.88  (1.68-18.48)^a^ | 9.63  (4.95) | 8.33  (2.13-24.51)^a^ | 154.3 (115.4) | 142.00  (10.04-436.3)^b^ |
| **Ceruloplasmin** | **(n= 17/92)** | | **(n= 10/11)** | | **(n= 5/6)** | | **(n= 114/125)** | | **(n= 40/46)** | |
| USpG (ng/mL) | 10.67 (5.94) | 9.10  (1.49-28.00)^a^ | 12.44 (5.94) | 12.41  (4.65-21.52)^a^ | 11.83 (10.18) | 6.76  (6.33-29.87)^a^ | 21.82 (23.18) | 18.56  (0.05-137.0)^a^ | 2.64  (2.90) | 1.49  (0.07-11.20)^a^ |
| Creatinine (ng/mg Cr) | 13.25 (9.99) | 11.37  (2.00-39.57)^a^ | 7.05  (4.17) | 5.81  (2.39-14.74)^a^ | 11.43 (16.45) | 4.74  (2.44-40.80)^a^ | 37.76 (48.15) | 23.65  (0.06-294.0)^a^ | 3.30  (7.33) | 0.98  (0.04-42.86)^a^ |
| Raw  (ng/ mL) | 14.02 (10.73) | 11.40  (1.30-40.20)^a,b^ | 35.77 (19.75) | 30.45  (12.80-78.00)^c^ | 20.43 (9.81) | 17.40  (11.20-36.60)^b^ | 13.97 (13.98) | 11.50  (0.10-68.50)^a,b^ | 4.19  (4.00) | 3.17  (0.10-16.70)^a^ |
| **PGFM** | **(n= 6/92)** | | **(n= 9/11)** | | **(n= 6/6)** | | **(n= 39/125)** | | **(n= 40/46)** | |
| USpG (ng/mL) | 9.65  (4.71) | 9.39  (3.72-17.74)^a^ | 22.61  (8.89) | 20.86  (12.10-37.30)^a,b^ | 35.68 (33.86) | 27.55  (9.37-101.8)^a,b^ | 6.66  (3.33) | 6.02  (2.06-13.96)^a^ | 55.37 (43.61) | 51.84  (2.36-163.1)^b^ |
| Creatinine (ng/mg Cr) | 14.03 (9.90) | 11.68  (5.01-32.87)^a^ | 13.00  (1.95) | 12.62  (10.66-16.26)^a^ | 20.09 (12.25) | 19.10  (6.58-41.75)^a^ | 12.65 (10.92) | 9.14  (3.75-52.38)^a^ | 46.67 (84.65) | 23.35  (3.48-536.5)^a^ |
| Raw  (ng/ mL) | 14.43 (11.11) | 11.93  (3.25-33.26)^a^ | 59.19 (24.24) | 53.71  (31.76-111.9)^a,b^ | 110.91 (137.76) | 79.78  (4.70-381.7)^b^ | 4.46  (2.93) | 3.64  (0.81-12.21)^a^ | 109.41 (93.40) | 119.64  (0.00-318.4)^b^ |
| **USpG** | **(n= 92/92)** | | **(n= 11/11)** | | **(n= 6/6)** | | **(n= 125/125)** | | **(n= 46/46)** | |
| USpG | 1.008 (0.003) | 1.008  (1.001-1.021)^a^ | 1.023 (0.005) | 1.022  (1.015-1.034)^c^ | 1.020 (0.009) | 1.021  (1.003-1.030)^b,c^ | 1.006 (0.003) | 1.005  (1.001-1.017)^a^ | 1.015 (0.012) | 1.012  (1.001-1.042)^b^ |
| **Cr** | **(n= 92/92)** | | **(n= 11/11)** | | **(n= 6/6)** | | **(n= 125/125)** | | **(n= 46/46)** | |
| Creatinine (mg/mL) | 0.67  (0.46) | 0.67  (0.00-2.63)^a^ | 5.91  (3.44) | 5.00  (2.75-13.23)^c^ | 4.79  (3.51) | 3.61  (0.27-9.14)^b,c^ | 0.42  (0.39) | 0.39  (0.00-2.87)^a^ | 3.48  (3.98) | 1.61  (0.00-13.42)^b^ |
| **Faeces (kg)** | **(n= 80/92)** | | **(n= 6/11)** | | **(n= 3/6)** | | **(n= 116/125)** | | **(n= 40/46)** | |
| Faeces  (kg) | 3.4  (0.9) | 3.5  (1.2-5.4)^a^ | 1.8  (1.2) | 1.5  (0.8-3.9)^a^ | 1.3  (0.3) | 1.4  (1.0-1.6)^a^ | 8.5  (2.0) | 8.9 (  1.6-12.6)^b^ | 3.0  (2.9) | 2.2  (0.2-11.2)^a^ |
| **Bodyweight** | **(n= 80/92)** | | **(n= 2/11)** | | **(n= 2/6)** | | **(n= 114/125)** | | **(n= 14/46)** | |
| Bodyweight (kg) | 107.1  (0.6) | 107.2  (104.6-108.3)^a,b^ | 104.9  (2.69) | 104.9  (103.0-106.8)^a,b^ | 102.6  (0.21) | 102.6  (102.4-102.7)^a^ | 114.0  (6.7) | 113.1  (103.0-126.5)^b,c^ | 117.3  (4.7) | 117.8  (110.0-123.4)^c^ |

Stdev = standard deviation; n = number of samples; USpG = urinary specific gravity; cr = creatinine. Different superscripts (a-d; ascending; horizontally) indicate significant differences for the respective metabolite levels between each defined reproductive period; Independent-Samples Kruskall Wallis test with post hoc Dunn’s comparison; significant if p < 0.05.
